# CD16 CAR-T cells enhance antitumor activity of CpG ODN-loaded nanoparticle-adjuvanted tumor antigen-derived vaccinevia ADCC approach

**DOI:** 10.1186/s12951-023-01900-8

**Published:** 2023-05-20

**Authors:** Xiaofei Zhang, Qin Hu, Xuesong He, Xinyue Cui, Zhaoyuan Liang, Li Wang, Xiongwei Deng, Ze Zhang, Wang Sheng, Xiaodong D. Han

**Affiliations:** 1 Beijing Key Laboratory of Microstructure and Properties of Solids, Institute of Microstructure and Property of Advanced Materials, Faculty of Materials and Manufacturing, Beijing University of Technology, Beijing, 100124 China; 2 Beijing International Science and Technology, Cooperation Base of Antivirus Drug, Department of Environment and Life Science, Beijing University of Technology, Beijing, 100124 China; 3 Department of Materials Science and Engineering and State Key Laboratory of Silicon Materials, Zhejiang University, Hangzhou, 310058 China; 4 Beijing Key Laboratory of Microstructure and Properties of Solids, Institute of Microstructure and Property of Advanced Materials, Beijing International Science and Technology, Cooperation Base of Antivirus Drug, Department of Environment and Life Science, State Key Laboratory of Silicon Materials, Beijing, 100005 Zhejiang 310058 China

**Keywords:** Vaccine, CpG ODN, Neoantigen, CD16, Immunotherapy

## Abstract

**Background:**

Combinatorial immunotherapy strategies for enhancing the responsiveness of immune system have shown great promise for cancer therapy. Engineered nanoformulation incorporated toll-like receptor (TLR) 9 agonist CpG ODN has shown more positive results in suppressing tumor growth and can significantly enhance other immunotherapy activity with combinatorial effects due to the innate and adaptive immunostimulatory effects of CpG.

**Results:**

In the present work, protamine sulfate (PS) and carboxymethyl β-glucan (CMG) were used as nanomaterials to form nanoparticles through a self-assembly approach for CpG ODN encapsulation to generate CpG ODN-loaded nano-adjuvant (CNPs), which was subsequently mixed with the mixture of mouse melanoma-derived antigens of tumor cell lysates (TCL) and neoantigens to develop vaccine for anti-tumor immunotherapy. The obtained results showed that CNPs was able to effectively deliver CpG ODN into murine bone marrow-derived dendritic cells (DC) in vitro, and remarkably stimulate the maturation of DC cells with proinflammatory cytokine secretion. In addition, in vivo analysis showed that CNPs enhanced anti-tumor activity of PD1 antibody and CNPs-adjuvanted vaccine based on the mixture antigens of melanoma TCL and melanoma-specific neoantigen could not only induce anti-melanoma cellular immune responses, but also elicit melanoma specific humoral immune responses, which significantly inhibited xenograft tumor growth. Furthermore, CD16 CAR-T cells were generated by expressing CD16-CAR in CD3^+^CD8^+^ murine T cells.

**Conclusion:**

Our results eventually showed that anti-melanoma antibodies induced by CNPs-adjuvanted TCL vaccines were able to collaborate with CD16-CAR-T cells to generate an enhanced targeted anti-tumor effects through ADCC (antibody dependent cell cytotoxicity) approach. CD16 CAR-T cells has thus a great potential to be an universal promising strategy targeting on solid tumor synergistic immunotherapy via co-operation with TCL-based vaccine.

**Supplementary Information:**

The online version contains supplementary material available at 10.1186/s12951-023-01900-8.

## Introduction

Tumor still represents one of the major causes of morbidity and mortality worldwide, and common treatment stratigies such as surgery, chemotherapy, and radiotherapy suffer from obvious limitations and problems [1, 2]. In the past decade, the rise of advanced anti-tumor immunotherapy has led to the dawn of tumor cures [3]. Currently, immune checkpoint inhibitors (anti-PD-1, and anti-PD-L1, anti-CTLA-4 antibodies), chimeric antigen receptor T (CAR-T) cell therapies and anti-tumor vaccines constitute the main immunotherapy strategies, which have been able to result in certain therapeutic effects and durable clinical responses in a subset of tumor types and patients [4–6]. Nevertheless, low or modest response rates and potential immune-related adverse events exist as the two major issues for anti-tumor immunotherapy for the majority of tumors [7–9]. Specially, melanoma is among the most sensitive of malignancies respond to immunotherapy. Despite the exciting clinical results of aPD1 therapy for improving the overall survival of melanoma, the objective response rate of aPD1 therapy in metastatic melanoma is merely 40% and the clinical efficacy of aPD1 still needs to be improved [10]. In addition, CAR-T cell therapy has illustrated the beneficial implications in hematological malignancies, while barriers in solid tumors cause CAR-T cells to become ineffective [11–13]. Therefore, there is an urgent need to improve current anti-tumor immunotherapies. As our understanding of immunotherapy continues to advance, it has become increasingly clear that the combinatorial immunotherapy strategies would be a widely applicable approach to make immunotherapy more effective [14, 15].

Recent years, anti-tumor therapeutic vaccines have been developed as an active strategy to specifically recognize and destroy malignant cells by inducing CTL responses and CD3^+^CD4^+^ T helper cell activation relying on tumor antigens. Tumor-associated antigens (TAAs) and tumor-specific neoantigen represent two major classes of tumor antigen [16–19]. Tumor cell lysate (TCL) provides a renewable pool of almost all the potential characterized and uncharacterized TAAs, while the responses generated from the TCL alone are always low due to its poor immunogenicity. Alternatively, identified peptide designed to elicit specific T cells against antigens selectively expressed by tumor cells are also served as important source of TAAs [20]. Of particular interest, tumor specific antigens (neoantigen) are attractive vaccine targets as they are not expressed in healthy tissues and are predicted to have strong major histocompatibility complex (MHC)-binding affinity [21–24]. Specially, neoantigen vaccines based on peptides are of primary importance over other approaches owing to robust safety profile and ease of manufacturing. Nevertheless, several concerns regarding peptide antigens including poor antigen presentation, low immunopotency, and a short half-life in vivo, have hampered their clinical translation. To overcome these challenges, adjuvant/delivery system and combinatorial immunotherapy strategies are developed to improve their immunogenic potency [25–27].

Last several years, the combination of anti-tumor immunotherapy with identified immunomodulator has become the hotspot of anti-tumor immunotherapy research and good therapeutic effect has been achieved in both preclinical and clinical studies [28–32]. Among them, toll-like receptor (TLR) agonists are a class of immunopotentiator adjuvants that effectively enhance immune responses, such as TLR-4 ligands, TLR-7/8 ligands and TLR-9 ligands, which can induce strong Th1 and CTL responses. Synthetic oligodeoxynucleotides (ODN) containing unmethylated CpG motifs have been identified as TLR-9 agonists, which could trigger innate and adaptive immunity. Until now, various CpG ODN has been studied in different stage clinical trials for cancer combinatorial immunotherapy [33–35]. For example, SD-101 was effective in 78% of patients with unresectable melanoma in combination with PD-1 inhibitors [36]. However, naked CpG ODN are clinically limited due to their rapid degradation and low efficiency of delivery to immune cells. To overcome these limitations, various non-viral nano-formulation based methods were developed to load and deliver CpG ODNs with positive results [37–42]. Previously, we have developed CpG ODN incorporated nanoformulation by using protamine sulfate (PS) and carboxymethyl β-glucan (CMG) through a self-assembly approach, which significantly increased the delivery efficiency of CpG ODN to DCs and presented excellent adjuvant function [37–42].

Having established CNPs as a CpG ODN-based immunomodulator or adjuvant platform, we thus hypothesis the enhanced therapeutic potential of combinational CNPs with currently used immunotherapy methods. Herein, we then explored single CNPs or combined antibody blockade of PD-1 (aPD-1) or combined with tumor-associated antigens of TCLs and neoantigens for treatment of murine melanoma carcinoma tumor. Based on the results that the assembled CNPs could effectively deliver CpG ODN to dendritic cells (DC), we found CNPs could significantly stimulate the maturation of DC cells and promote proinflammatory cytokine secretion. In addition, CNPs could improve the therapeutic effects of aPD-1 with combinatorial effects and significantly prolong the survival rate of B16F10 tumor-bearing mice. Intriguingly, further combination of TCL and neoantigen peptides with CNPs could also promote the expansion of cytotoxic T cells, enhance immune responses and significantly inhibit tumor growth and prolong the survival of tumor-bearing mice. Moreover, the serum extracted with immunized mice could enhance the efficacy of CD16-CAR-T cells for improved melanoma carcinoma tumor therapy. Collectively, we reported a novel synergistic immunotherapy strategy by combining tumor specific antigens-based nano-adjuvanted vaccine with PD1 antibody and CD16 CAR-T immune cell treatment for effective targeted anti-tumor immunotherapy.

## Results and discussion

### Preparation, physicochemical characterization of CNPs and in vitro regulation of BMDCs by CNPs

**Scheme 1:**
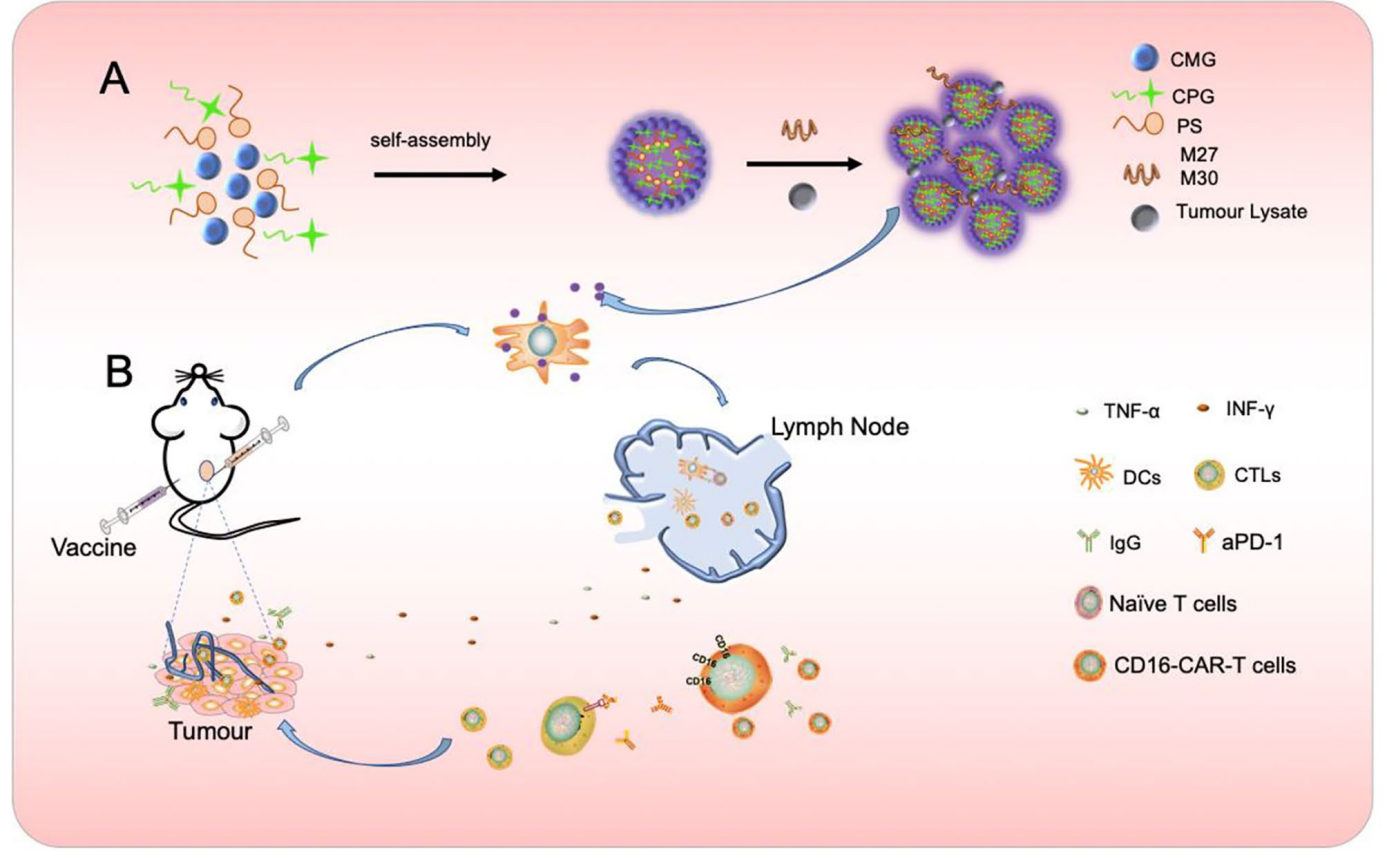
Formation of CNPs nanoparticles and synergistic anti-tumor immunotherapy strategy based on vaccine, anti-PD1 antibody and CD16 CAR-T cells. A CpG ODN was encapsulated by CMG and PS to form CpG ODN –loaded nanoparticle (CNPs) through self-assembly approach. Vaccines were generated by mixing CNPs with tumor-derived antigens, such as TCL and neoantigens or the mixture of TCL and neoantigens, respectively. B Vaccine-induced anti-tumor CTL and tumor-specific antibodies, which directed CD16 CAR-T to target tumor cells through ADCC approach and the targets of Anti-PD1 antibody

**Fig. 1:**
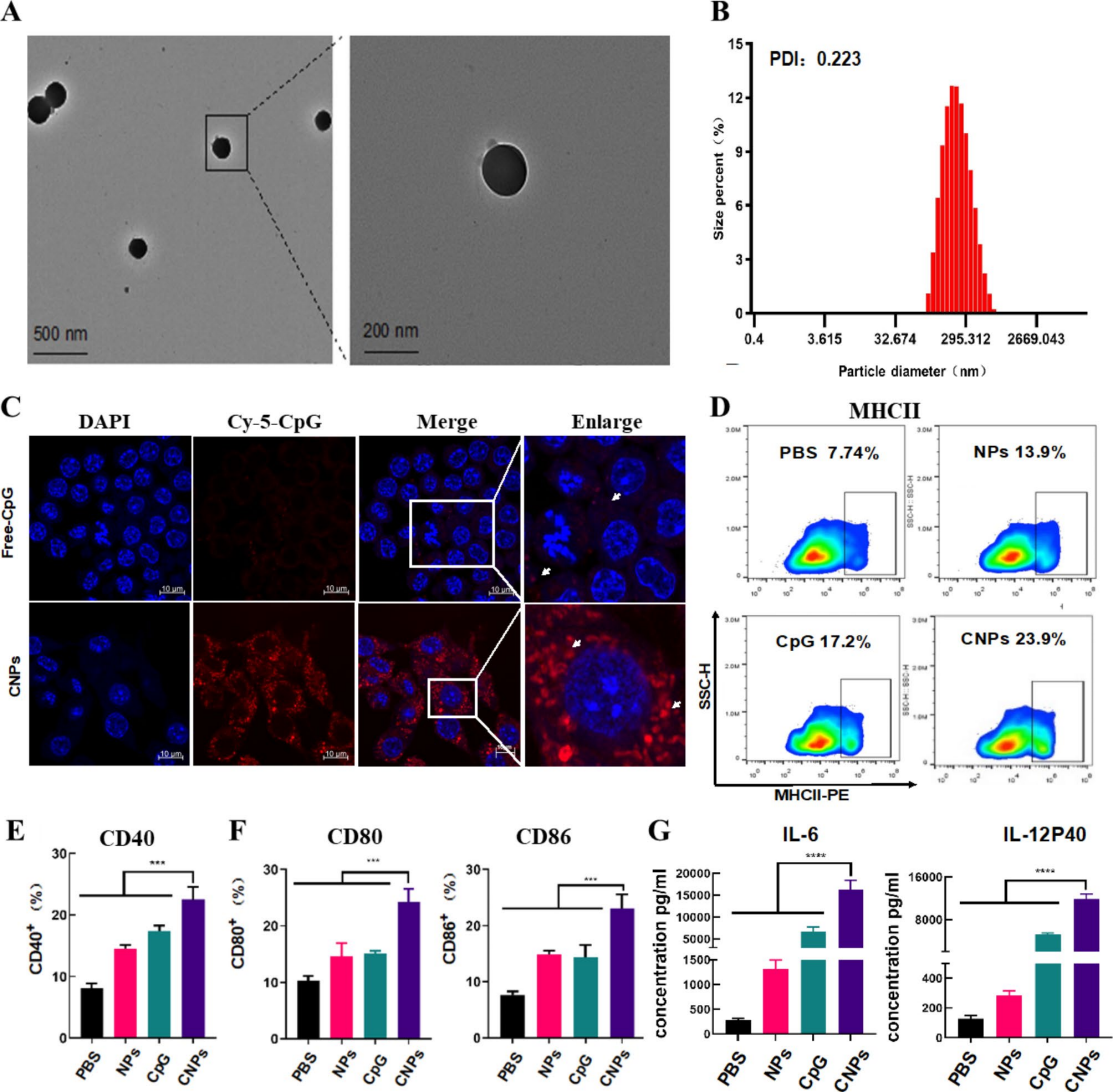
Characterization of CNPs, Intracellular uptake of CpG ODN by DCs and activation of DCs. (**A**) TEM (transmission electron microscopy) imaging of CNPs. (**B**) DLS (dynamic light scattering) analysis. (**C**) Fluorescent imaging of intracellular uptake of FITC-labeled CpG ODN by DCs. (**D**) Expression of MHC-II analyzed by flow cytometry. (**E**) Expression of CD40 analyzed by flow cytometry. (**F**) Expression of CD80 and CD86 analyzed by flow cytometry. (**G**) Secretion of IL-6 and IL12P40. Data are presented as means ± s. d. Statistical significance was calculated by one-way analysis of variance (ANOVA). (n = 3), **, P < 0.01 ***, P < 0.001

Self-assembled polyelectrolyte nanocomplexes (SPECs) typically formed by oppositely charged polyelectrolytes, represents a facile way to generate self-assembled nanocomplexes depending on the building blocks and parameters. Electrostatic interactions between oppositely charged polyelectrolytes of protamine sulfate (PS) and carboxymethyl β-glucan (CMG) led to the formation of CMG-PS nanoparticles (NPs) and CpG ODN was subsequently incorporated into CMG-PS NPs to generate CpG ODN-loaded nanocomplexes (CNPs) through a self-assembly approach (scheme 1). The mass ratio of PS, CMG and CpG ODN at 1:2:0.0375 was established and used in this experiment to generate CNPs with optimal size, zeta potential and CpG loading efficiency (Table. S1). TEM analysis showed that CNPs exhibited spherical morphology with homogenous size distribution without aggregation (Fig. 1A). The PDI of CNPs was 0.223 (Fig. 1B). However, smaller diameter of CNPs was detected by TEM analysis than that detected by DLS measurements (Fig. 1B), which could be ascribed to the distinction between the dehydration of CNPs corona in dry state in TEM analysis and the hydrodynamic swelling state of the samples suspended in aqueous media in DLS measurements. In addition, the zeta potential of the nanocomplexes changed from negative − 19.9 mV charge to -23.1 mV charge with the loading of CpG ODN (Fig. S1). Furthermore, serum stability of free CpG ODN and CpG ODN in the formulation of CNPs was evaluated. The results showed that free CpG ODN started to degrade gradually after 24 h and CpG ODN completely degraded after 24 h. In contrast, the CpG ODN in the formulation of CNPs was stable and could be protected from degradation (Fig. S2). In addition, we found that the release efficacy of CpG-ODN could reach nearly 50% in 70 h (Fig. S3).

The cell cytotoxicity of CNPs was detected in B16F10 cells and normal cell line (HUVEC) by measuring cell viability, in which negligible toxicity of CNPs was found upon CNPs treatment (Fig. S4). The obtained results indicated a good biocompatibility of CNPs and the low cytotoxicity of CNPs is correlated with the high biocompatibility of PS and CMG. DCs are the most potent professional antigen present cells (APCs) that play a critical role in mediating innate response and inducing adaptive immune response [43–45]. Next, we quantified the cellular phagocytosis efficiency of free CpG ODN and CpG ODN-loaded CNPs in bone marrow derived dendritic cells (BMDCs) by laser scanning confocal microscope analysis (LSCM). CpG ODN was labeled with Cy-5 fluorescence (Cy-5-CpG) for direct measurement of the delivery efficiency of CpG ODN into DCs by fluorescence imaging. Isolated immature BMDCs were incubated with free Cy-5-CpG and Cy-5-CpG encapsulated in CNPs for 24 h, respectively. As shown in Fig. 1C, no or little fluorescent signal was detected in the cells incubated with free CpG ODN. In contrast, strong fluorescent signals were found in CNPs-treated cells, suggesting that CpG ODN can be efficiently delivered into DC cells upon PS and CMG-mediated nano-formulation (Fig. 1C).

### In vivo accumulation of CNPs into dLNs and CNPs enhanced anti-tumor effects of aPD-1 antibody in B16F10 tumor-bearing mice

Targeting delivery of CpG ODN to inguinal LNs (lymph node) is an important element in determining the efficiency of triggering innate and adaptive immunity in vivo since the abundant DCs at LNs [46, 47]. We subsequently evaluated the distribution of CNPs to draining lymph node in mice after subcutaneous injection, in which C57BL/6 mice were subcutaneously injected with Cy-5 labeled free CpG ODN, CNPs and PBS, and fluorescent signals were monitored from isolated LNs at 6 h, 12 h, 24 and 72 h upon injection. The obtained data showed that no and little fluorescent signals were detected in PBS- and CPG ODN-treated groups. In contrast, strong fluorescent signals were found in CNPs-treated group 24 h after injection, indicating that CPG ODN can be efficiently delivered into LN upon encapsulation by PS@CMG-based nanoparticles. Potent fluorescent signals were equally detected in LN 72 h after injection, suggesting a sustained delivery of CPG ODN in LN, which is obviously favorable for long-lasting activation of immune response (Fig. S6).

**Fig. 2:**
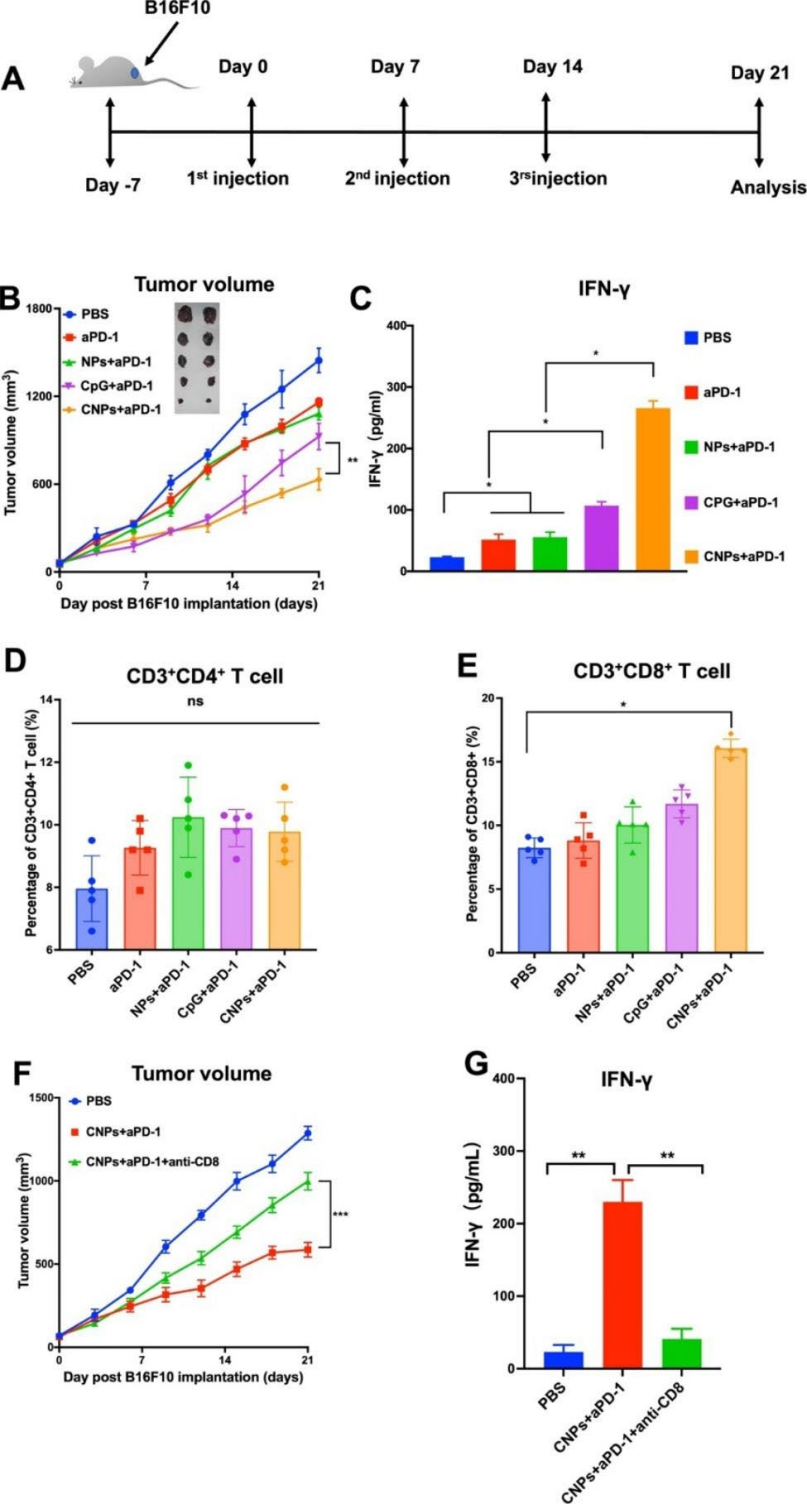
CNPs enhanced anti-tumor activity of anti-PD1 antibody (aPD1). (**A**) Treatment and analysis schedule. (**B**) Tumor growth curve. (**C**) INF-γ secretion in sera. (**D**) The infiltration of CD3^+^CD4^+^ T cells in tumor tissues. (**E**) The infiltration of CD3^+^CD8^+^ T cells in tumor tissues. (**F**) Tumor growth curves. (**G**) INF-γ secretion in sera. The data were presented as means ± s.d. Statistical significance was calculated by one-way analysis of variance (ANOVA). (n = 5), *, P < 0.05; **, P < 0.01

Immune checkpoint inhibitor such as aPD1 has been widely used in anti-tumor therapy, even though the therapeutic efficacy of PD1 blockage is variable and dependent on tumor types [48]. Encouraged by the above efficient immune regulatory effects of CNPs on DCs in vitro, we therefore explored the combination therapeutic effects of the CNPs with aPD1 in B16F10 tumor model. Mice were divided into 5 groups, including PBS-, aPD1-, NPs + aPD1- (empty NPs combined with aPD1), CpG + aPD1- (free CPG ODN combined with aPD1) and CNPs + aPD1-treated groups. Injection was carried out for three times with 7 days interval, while the tumor size reached about 100 mm^3^. The injection schedule was displayed in Fig. 2A. The results showed that, compared to the PBS control, reduced tumor sizes were found in aPD1-treated mice, indicating a tumor inhibition roles of aPD1, which is correlated with the previous report [48]. Few smaller tumor sizes were found in both NPs + aPD1- and CpG + aPD1-treated groups, in comparison with those in aPD1-treated group, suggesting that both NPs and free CPG ODN were able to slightly improve tumor inhibitory effects of aPD1. However, significant tumor growth inhibition was found in CNPs + aPD1-treated group, compared to the other groups, illustrating that the increase of anti-tumor effects in CNPs + aPD1-treated group may be attributed to the encapsulation of CpG ODN in CNPs (Fig. 2B). The survival rates of mice were show synergistic tumor inhibitory in Fig. S7, in which the highest survival rate was found in CNPs + aPD1-treated group, compared to the other groups. However, the mice in the PBS-treated group died 25 days post treatment. Sera were collected upon sacrifice of the mice for IFN-γ examination. The results showed that the average concentration of IFN-γ in CNPs + aPD1-treated group was much higher than the other groups and about 2.5 times higher than that in free CpG + aPD1-treated group (Fig. 2C), revealing a significantly increased activity of CPG ODN in immune response activation upon nanoparticle encapsulation, which is consistent with the above in vitro data. Furthermore, we analyzed tumor infiltrating T cells from isolated tumor tissues. The obtained results showed that, compared to PBS control group, no significant increase in CD3^+^CD4^+^ tumor infiltrating T cells was found in aPD1-, NPs + aPD1-, CPG + aPD1- and CNPs + aPD1-treated groups. However, the CD3^+^CD8^+^ tumor infiltrating T cells were significantly increased in the CNPs + aPD1-treated group, compared to those in the other groups (Fig. 2D-E), indicating that CNPs could notably trigger the infiltration of CD3^+^CD8^+^ T cells in tumor tissues. Furthermore, CD3^+^CD8^+^ T cell deletion experiments were carried out to address the relationship of tumor infiltrating CD3^+^CD8^+^ T cell with tumor growth, in which CD3^+^CD8^+^ T cells were specifically removed by the treatment of anti-CD8 monoclonal antibody. The data showed that, compared with PBS control group, tumor growth was significantly suppressed in CNPs + aPD1-treated group. However, reduced tumor growth inhibition was observed in CNPs + aPD1 + anti-CD8 group, in which CD3^+^CD8^+^ T cells were especially removed by anti-CD8 monoclonal antibody. The obtained results demonstrated that the infiltration of CD3^+^CD8^+^ T cells in tumor tissues played a pivotal role in tumor growth inhibition and tumor suppression was remarkably reduced upon the removal of CD3^+^CD8^+^ T cell by the administration of anti-CD8 monoclonal antibody (Fig. 2F). The secretion of INF-γ in sera was further examined upon anti-CD8 antibody treatment, the data demonstrated that, compared with PBS group, INF-γ secretion was dramatically increased in CNPs + aPD1-treated group, which is correlated with the above data that tumor infiltrating CD3^+^CD8^+^ T cell is remarkably increased in CNPs + aPD1-treated group. However, INF-γ secretion was notably diminished in CNPs + aPD1 + anti-CD8-treated group upon anti-CD8 antibody administration. INF-γ is predominantly produced by CD3^+^CD8^+^ T cells. The reduction of INF-γ secretion in CNPs + aPD1 + anti-CD8-treated group revealed that CD3^+^CD8^+^ T cells were indeed specifically eliminated by anti-CD8 antibody (Fig. 2G).

### CNPs enhanced the immunogenicity of tumor-derived antigens

**Fig. 3:**
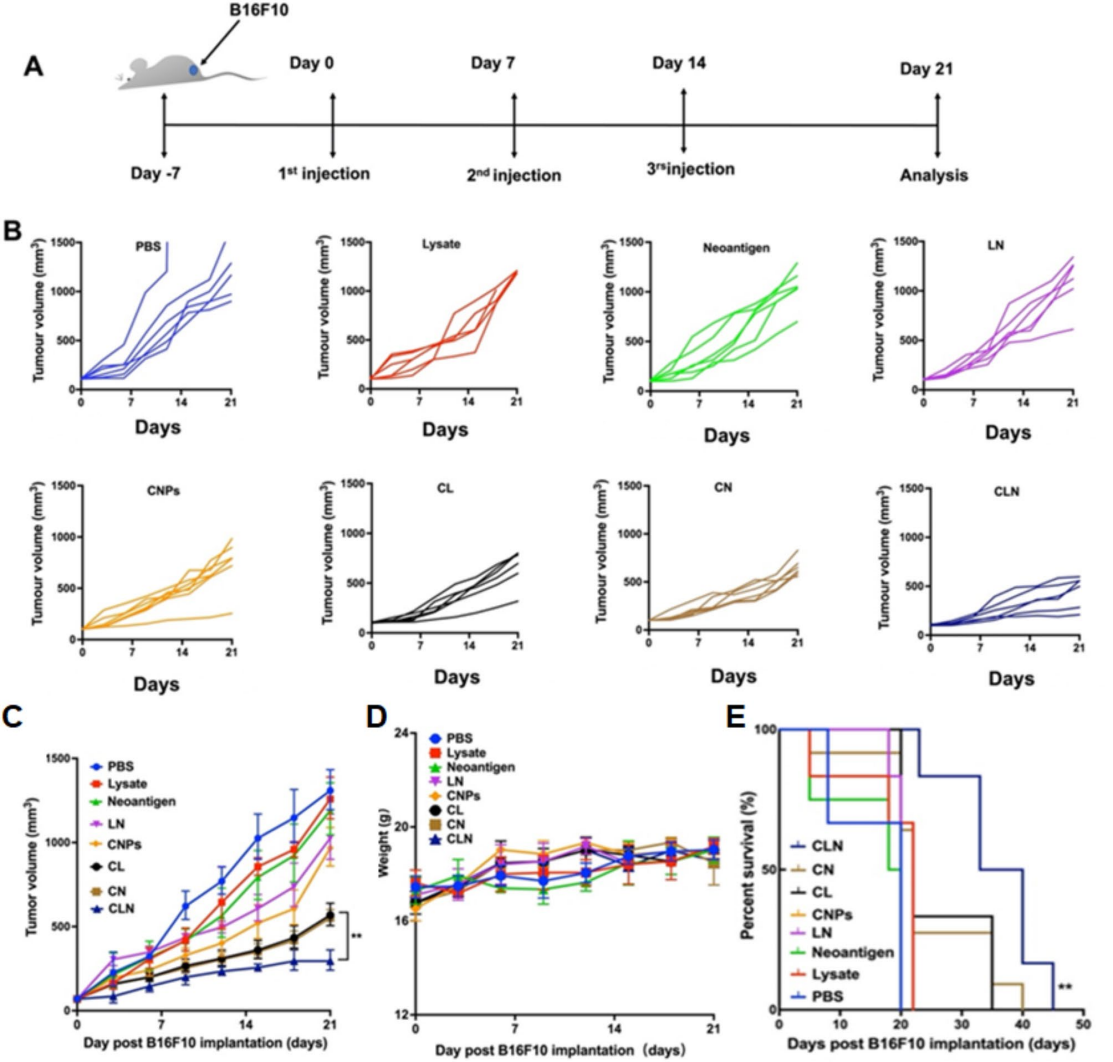
Analysis of tumor growth, body weights and survival rates. (**A**) Treatment schedule. (**B**) Individual tumor growth curves of each animal treated with different substrates, including PBS, lysate, neoantigens, LN, CL, CN and CLN. (**C**) Tumor volumes were measured at indicated time-points and shown as average of duplicate measurements with standard error bars (n = 6 for each group). (**D**) The body weights of mice were measured at indicated time-points and shown as average of duplicate measurements with standard error bars (n = 6 for each group). (**E**) Survival curves of mice after various treatments

Tumor antigens are currently divided into two major classes, including TAA (tumor associated antigen) and TSA (tumor specific antigen). Tumor cell lysate (TCL) results from artificial lysis of tumor cells and is a mixture of proteins derived from tumor cells, which is a rich source of TAA. Neoantigens are generated by genetic mutations in tumor cells and are a kind of TSA, which are only expressed in tumor cells. Two neoantigen peptides named as M30 (MHC II restricted) and M27 (MHC I restricted) have previously been reported in B16F10 cells as TSAs [47, 49]. TCLs and neoantigen peptides derived B16F10 cells were therefore adopted as tumor antigen resources and mixed with CNPs to evaluate their immunological responses and anti-tumor effects upon subcutaneous immunization. Mice were thus divided into 8 groups for vaccination, including PBS group, lysate group (TCL group), neoantigen group (M27 + M30, mixture of two peptides), LN group (lysate + neoantigens), CNPs group, CL group (CNPs + lysate), CN group (CNPs + neoantigens), CLN (CNPs + lysate + neoantigens). Immunization schedule was exhibited into Fig. 3A. Mice were inoculated every week for three consecutive weeks, while tumor size reached about 100 mm^3^. Tumor volume was measured every three days to monitor tumor growth. The observed data showed that tumor volumes in lysate- and neoantigen-treated groups were similar to those in PBS group, indicating both TCL and neoantigens have no or low immunogenicity, which is consistent with previous reports [47, 49, 50] (Fig. 3B-C, S8). Slightly reduced tumor volumes were observed in LN- and CNPs-injected groups, respectively, compared to the PBS group, suggesting that antigenic immunogenicity can be enhanced by the mixture of TCL with neoantigens, and CNPs administration alone is able to generate anti-tumor effects in vivo, correlated with the above data (Fig. 3B-C, S8). Obviously reduced tumor volumes were obtained in CL- and CN-treated groups, compared to those of PBS-, lysate- and neoantigen-treated groups, suggesting the mixture of CNPs is capable of dramatically enhancing the immunogenicity of TCL and neoantigen peptides, which both induced remarkable anti-tumor immune responses and inhibited tumor growth (Fig. 3B-C, S8). However, the highest inhibition of tumor growth was found in CLN-treated group, demonstrating that CLN administration induced the most potent anti-tumor immune responses with the strongest tumor growth inhibitory activity, which is consistent with the above data that antigenic immunogenicity can be enhanced by the mixture of TCL with neoantigens (Fig. 3B-C, S8). Our results thus exhibited that antigenic immunogenicity can be enhanced by the mixture TCL with neoantigens and CNPs played a pivotal role in elicitation of potent anti-tumor immune responses upon mixed with tumor antigens. In addition, there was no significant difference in mouse body weight among all groups (Fig. 3D), suggesting a good biocompatibility of vaccination components. Moreover, H&E (Hematoxylin and eosin) staining of the major organs (heart, liver, spleen, lung, and kidney) showed that no obvious pathological abnormalities were found in the main organs of mice treated with CLN compared with the PBS groups (Fig. S9), which is correlated with body weight measurement results. However, the survival rate of mice was remarkably improved in the CLN group compared to the other groups, which is accordant with tumor growth data (Fig. 3E). These results provide compelling evidence that CLN inoculation resulted in significantly increased anti-tumor immune responses with a good biological safety.

### Analysis of inoculation-induced cellular immunity, cytokine secretion and memory T cell responses

**Fig. 4:**
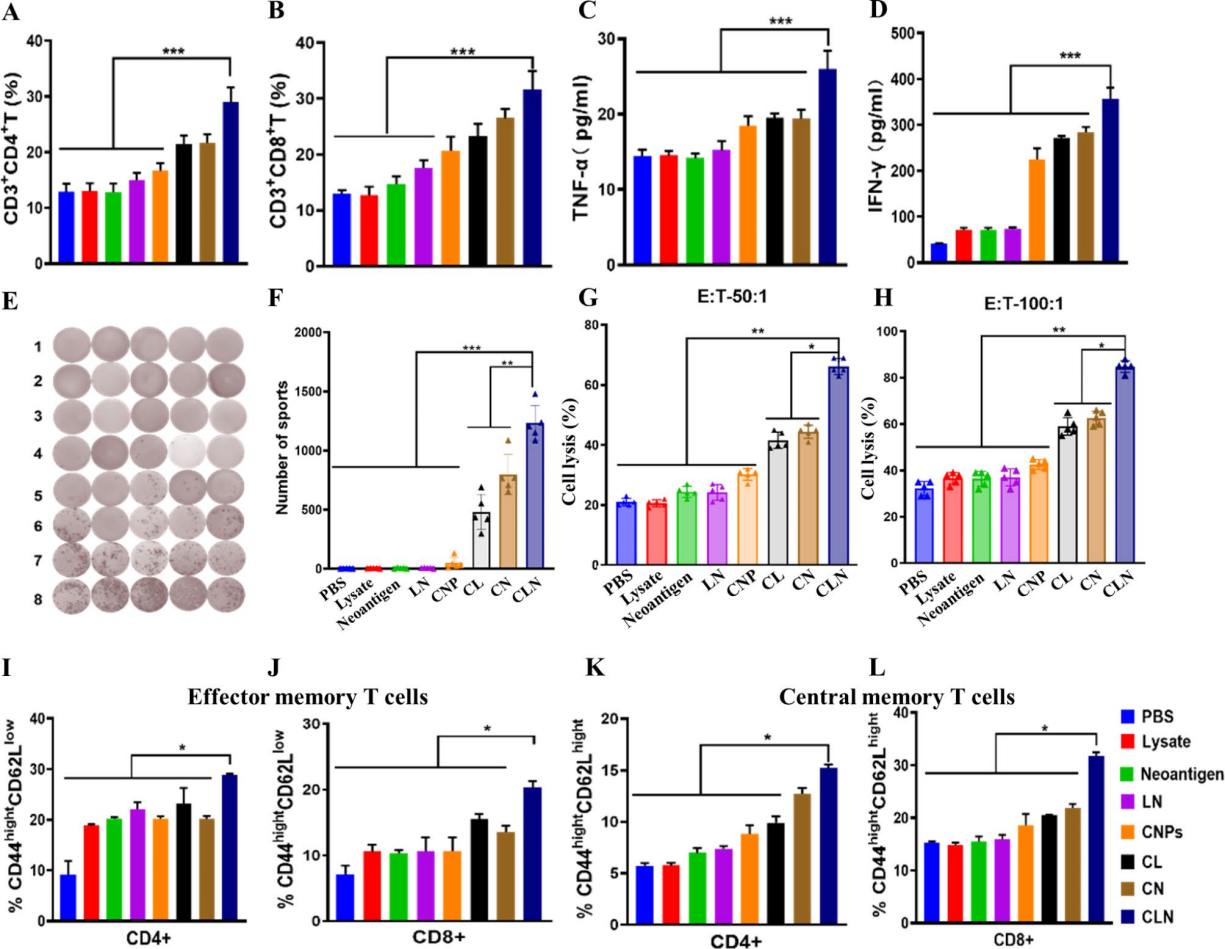
Flow cytometry and elispot analysis and CTL assays. (**A**) The percentage of CD3^+^CD4^+^ T cells infiltrated in tumor tissues. (**B**) The percentage of CD3^+^CD8^+^ T cells infiltrated in tumor tissues. (**C**) The secretion of TNF-α in serum samples. (**D**) The secretion of INF-γ in serum samples. (**E**) The imaging results of elispot analysis on INF-γ expression in isolated spleens. (**F**) INF-γ expression in isolated spleens. (**G**) CTL data with E:T 50:1. (**H**) CTL data with E:T 100:1. (**I**) CD4^+^CD44_high_CD62L_low_ effector memory T cells. (**J**) CD8^+^CD44_high_CD62L_low_ effector memory T cells. (**K**) CD4^+^CD44_high_CD62L_high_ central memory T cells. J CD8^+^CD44highCD62Lhigh central memory T cells. The data were presented as means ± s. d. Statistical significance was calculated by one-way analysis of variance (ANOVA). (n = 5), *, P < 0.05; **, P < 0.01

Cellular immunity plays a central role in anti-tumor immune responses. The percentages of CD3^+^CD4^+^ and CD3^+^CD8^+^ T cells were subsequently evaluated in isolated tumor tissues, bloods and spleens. The analyses in tumor tissues showed that the percentages of CD3^+^CD4^+^ T cells in lysate- and neoantigen-challenged groups were similar to those in PBS group, suggesting the low immunogenicity of TCLs and neoantigens (Fig. 4A, S10). Compared to those in PBS-, lysate- and neoantigen-challenged groups, slightly increased CD3^+^CD4^+^ T cell proportion were detected in LN- and CNPs-challenged groups, which is consistent with enhanced immunogenicity of LN and the adjuvant activity of CNPs (Fig. 4A, S10). Compared with those in lysate- and neoantigen-challenged groups, the percentages of CD3^+^CD4^+^ T cells were obviously augmented in CL- and CN-challenged groups, indicating the presence of CNPs is able to definitely increase immune responses against low immunogenic antigens (Fig. 4A, S10). Significantly increased CD3^+^CD4^+^ T cell proportion was found in CLN-challenged group, suggesting that the mixture of TCL with neoantigens is an optimal tumor antigen candidate for elicitation of potent anti-tumor immune responses (Fig. 4A, S10). Furthermore, the measurement of CD3^+^CD8^+^ T cell infiltration in tumor tissues showed that comparable proportions of CD3^+^CD8^+^ T cell were exhibited in lysate- and neoantigens-challenged groups in comparison with PBS group, correlated with the poor immunogenicity of TCL and neoantigen peptides (Fig. 4B, S10). Modest increases of CD3^+^CD8^+^ T cell proportion were shown in LN- and CNPs-challenged groups, which is in correlation with an improved immunogenicity of LN than lysate and neoantigens and adjuvant activity of CNPs, respectively (Fig. 4B, S10). Obvious increases of CD3^+^CD8^+^ T cell proportion in CL- and CN-challenged groups compared to lysate- and neoantigen-challenged groups, which is consistent with a central role of CNPs adjuvant in elicitation of dramatically up-regulated immune response to poor immunogenic antigens (Fig. 4B, S10). Significant increase of CD3^+^CD8^+^ T cell proportion was demonstrated in CLN-challenged group, suggesting a great approach of the mixture of TCL with neoantigens as tumor antigen resource for potent anti-tumor immune response elicitation (Fig. 4B, S10). However, no obvious changes in the percentages of CD3^+^CD4^+^ and CD3^+^CD8^+^ T cells were identified in bloods and spleens (data not shown).

The secretion level of TNF-α and IFN-γ were detected in the serum samples. The results exhibited that TNF-α secretion levels in CNPs-contained groups, including CNPs-, CL-, CN- and CLN-challenged groups, were higher than those groups without CNPs, including PBS-, lysate-, neoantigen- and LN-challenged groups, indicating an important role of CNPs in triggering immune response. However, TNF-α secretion was significantly increased in the CLN-challenged group compared to the other groups, which is consistent with the above analysis data of T cell percentage. (Fig. 4C). In addition, IFN-γ secretion levels were significantly up-regulated in CNPs-contained groups including CNPs-, CL-, CN- and CLN-challenged groups than those in groups without CNPs including PBS-, lysate-, neoantigen- and LN-challenged groups, suggesting the pivotal role of adjuvant activity of CNPs in the induction of immune responses (Fig. 4D). However, IFN-γ secretion level was tremendously up-regulated in CLN-challenged groups, which is consistent with the above T cell proportion data (Fig. 4D).

Splenocytes from mice immunized with PBS, lysate, neoantigen, LN, CNPs, CL, CN and CLN were harvested and stimulated with CLN. After incubation for 48 h, culture supernatants were analyzed for IFN-γ secretion by Elispot. The immunization of lysate, neoantigen, CLN and CNPs were not able to elite obvious IFN-γ response, as well as PBS (Fig. 4E, F). In contrast, significant IFN-γ secretion occurred following CLN stimulation in CL-, CN- and CLN-immunized groups, indicating tumor-related antigens and adjuvant are both indispensable for IFN-γ response elicitation. However, the highest IFN-γ response was detected in CLN-immunized group, suggesting CLN is more potent than tumor cell lysate and tumor-derived neoantigen in elicitation tumor specific immune responses (Fig. 4E, F).

Immunization-induced cytotoxic activity of T cells (CTL) against B16F10 tumor cells was examined by in vitro assays. Isolated effector T cells from the spleens of immunized mice were incubated with B16F10 tumor cells at two different cell number ratios: 50:1, E:T (Effector T cell:Tumor B16F10 cell) and 100:1 E:F. The obtained results showed that the CTL-mediated tumor cell lysis in lysate-, neoantigen-, LN- and CNPs-immunized groups was comparable to that in control PBS-group in both 50:1 and 100:1 E:T ratios (Fig. 4G, H). In contrast, increased tumor cell lysis was observed in CL-, CN- and CLN-immunized groups in all ratios (Fig. 4G, H), suggesting that immunization of CL, CN and CLN is able to induce anti-tumor CTL response and inoculation of lysate, neoantigen and LN is unable to elicit CTL response without CNPs adjuvant. However, the most potent anti-tumor CTL response was found in CLN-immunized group and CNPs alone is unable either to promote anti-tumor CTL-response without the presence of tumor-derived antigens.

For memory T cell analysis, CD44_high_CD62L_low_ and CD44_high_CD62L_high_ were used as makers to define the proportions of effector memory T cells and central memory T cells. The data showed that, in comparison with PBS group, CD4^+^ effector memory T cells with CD44_high_CD62L_low_ phenotypes were variably up-regulated in all inoculation groups, and the highest up-regulation was found in CLN-challenged group, indicating a potent ability of CLN in activation of CD4^+^ effector memory T cells (Fig. 4I). In addition, we found that CD8^+^ effector memory T cells with CD44_high_CD62L_low_ phenotype were variably up-regulated in all inoculation groups, compared to PBS group, and the highest up-regulation was exhibited in CLN-challenged group, suggesting a powerful ability of CLN in activation of CD8^+^ effector memory T cells (Fig. 4J, S11).

For the analysis of the proportions of CD4^+^ central memory T cells with CD44_high_CD62L_high_ phenotypes, the obtained results demonstrated that gentle up-regulation of the proportions of CD4^+^ central memory T cells was observed in neoantigen-challenged group, compared to lysate-challenged group, which showed similar proportion to PBS group. The data suggested that, compared to tumor cell lysates, neoantigen challenge was able to gently increase the proportions of CD4^+^ central memory T cells (Fig. 4K). Otherwise, the proportion of CD4^+^ central memory T cells in LN-challenged group was similar than that in neoantigen-challenged group, which is consistent with that LN is made of the mixture of neoantigen and tumor cell lysate (Fig. 4K). Compared to PBS groups, the proportion of CD4^+^ central memory T cells was up-regulated in CNPs-challenged group, indicating that CNPs alone is able to modulate CD4^+^ central memory T cell proportion (Fig. 4K). In addition, obvious up-regulations of the proportion of CD4^+^ central memory T cells were found in CL-, CN- and CLN-challenged groups, compared to lysate-, neoantigen-, and LN-challenged groups, respectively, suggesting a central role of CNPs in the up-regulation of the proportion of CD4^+^ central memory T cells. However, the highest proportion was found in CLN-challenged group, suggesting the most potent immune regulatory activity of CLN formulation (Fig. 4K). Moreover, the results of the analysis of the proportion of CD8^+^ central memory T cells were similar to those of the proportion of CD4^+^ central memory T cells, in which the most significant up-regulation of the proportion of CD8^+^ central memory T cells was found in CLN-challenged group, which is correlated with the most potent immune regulatory activity of CLN formulation (Fig. 4L).

### CL induced tumor-specific humoral immune responses

**Fig. 5:**
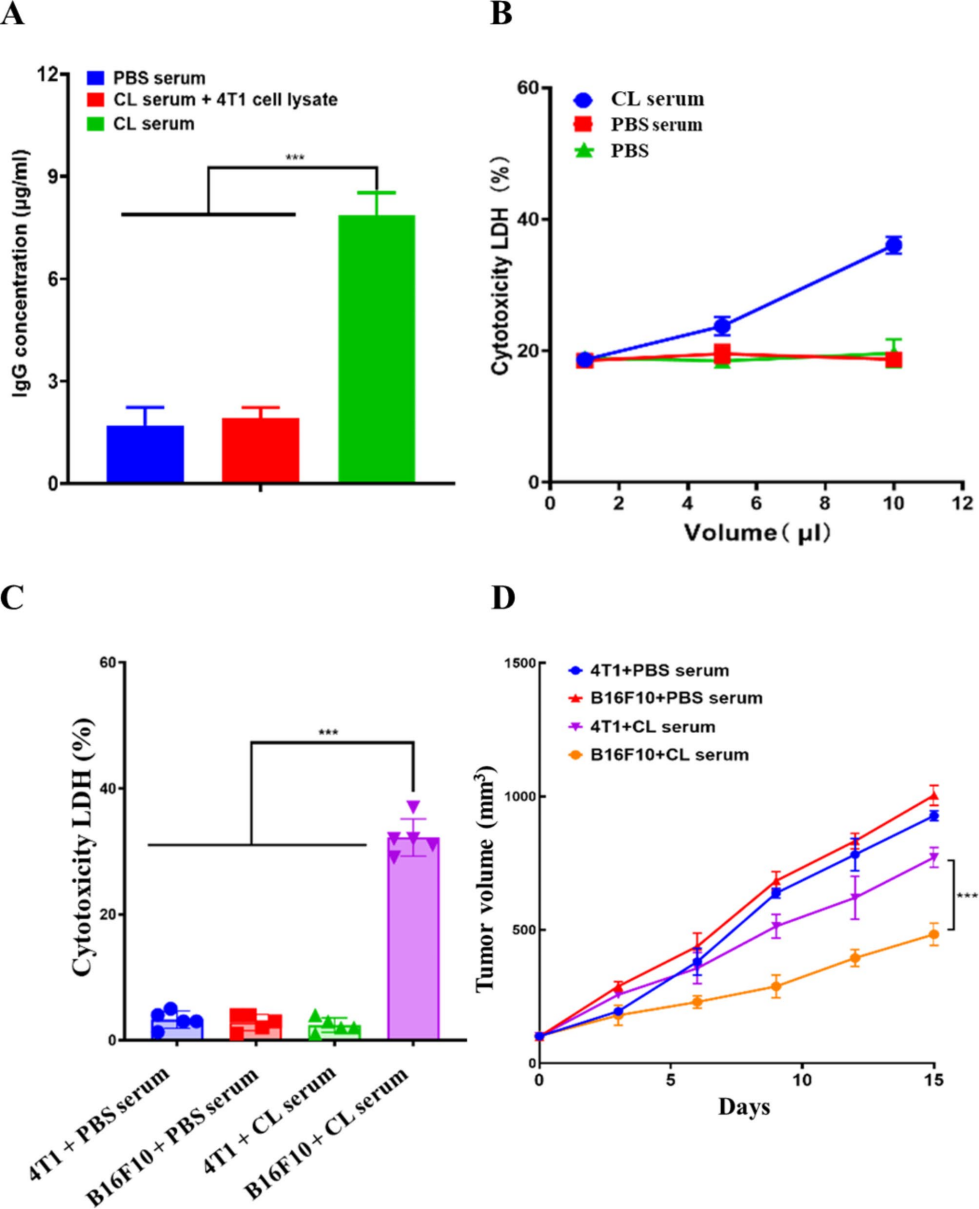
Detection and anti-tumor activity of CL-elicited humoral immune responses. (**A**) The concentration of Ig antibodies in various serum samples. (**B**) Cytotoxic effects of serum samples on B16F10 tumor cells. (**C**) Cytotoxic effects of serum samples on B16F10 and 4T1 tumor cells. (**D**) Anti-tumor growth activity of various serum samples. The data were presented as means ± s. d. Statistical significance was calculated by one-way analysis of variance (ANOVA). (n = 5), ***p < 0.001

TCL is a rich resource of TAA and TSA. We were therefore interested in investigating whether TCL as tumor-derived antigens were able to induce anti-tumor humoral immune responses in the presence of CNPs adjuvant. TCL of B16F10 cells was coated at the surfaces of 96-well microplates and incubated with sera collected from PBS- and CL-treated mice for quantitative antibody analysis, respectively. TCL of 4T1 cells coated at the surfaces of 96-well microplates was equally incubated with sera collected from CL-immunized mice to investigate cross-reaction of the isolated sera. The results showed that humoral responses were especially detected in CL-immunized mice and not found in PBS-treated mice as well as 4T1 cell lysates, suggesting that PBS treatment was unable to induce melanoma antigens-specific humoral responses (Fig. 5A). In contrast, immunization of CL was able to induce melanoma antigens-specific humoral responses, which did not cross-react with 4T1 cell lysate antigens (Fig. 5A).

Melanoma antigens-specific humoral responses were detected in CL-immunized mice. We were subsequently interested in studying whether anti-CL sera had anti-melanoma activity. Cultured B16F10 cells w ere incubated with variable volumes of anti-CL sera. B16F10 cells treated with PBS and sera isolated from PBS-treated mice were used as controls. The results showed that B16F10 cell death was particularly found in anti-CL sera-treated samples and no cell death was detected in samples treated with PBS and sera isolated from PBS-treated mice, suggesting an anti-tumor effect of anti-CL sera (Fig. 5B). Moreover, the increase in the volume of anti-CL sera is correlated with the increase in cell death, indicating that anti-CL sera treatment trigger B16F10 cell death in a dosage-dependent manner (Fig. 5B). In addition, 4T1 cells were incubated with the sera isolated from CL-immunized and PBS-treated mice, respectively, and compared with B16F10 cells incubated with the sera isolated from CL-immunized and PBS-treated mice. The obtained results showed that no cell death was detected in 4T1 cells incubated with either the sera isolated from CL-immunized or PBS-treated mice, as well as B16F10 cells incubated with the sera isolated from PBS-treated mice. In contrast, cell death was specifically found in B16F10 cells incubated with the sera isolated from CL-immunized mice (Fig. 5C). The data suggested that anti-CL sera was unable to induce the cell death of 4T1 cells, which were correlated with antigenic specificity of anti-CL sera that specifically interacted with B16F10 cell-derived antigens.

Xenograft mice were used to investigate anti-tumor activity of anti-CL sera in vivo. B16F10 and 4T1 cells were subcutaneously injected in nude mice, respectively. Anti-CL sera were administrated through tail vein injection at indicated time points. The sera isolated from PBS-treated mice were used as controls. The data showed that, compared to B16F10 and 4T1 cells-transplanted mice treated with PBS sera, anti-CL sera treatment significantly inhibited B16F10 cells-mediated malignant growth, which did not suppress 4T1 cells-promoted tumor growth (Fig. 5D). The obtained results indicated immunization CL is able to induce melanoma specific humoral response, which harbored specific anti-melanoma activity in vitro and in vivo.

### CD16 CAR-T enhanced anti-tumor activity of anti-CL sera

**Fig. 6:**
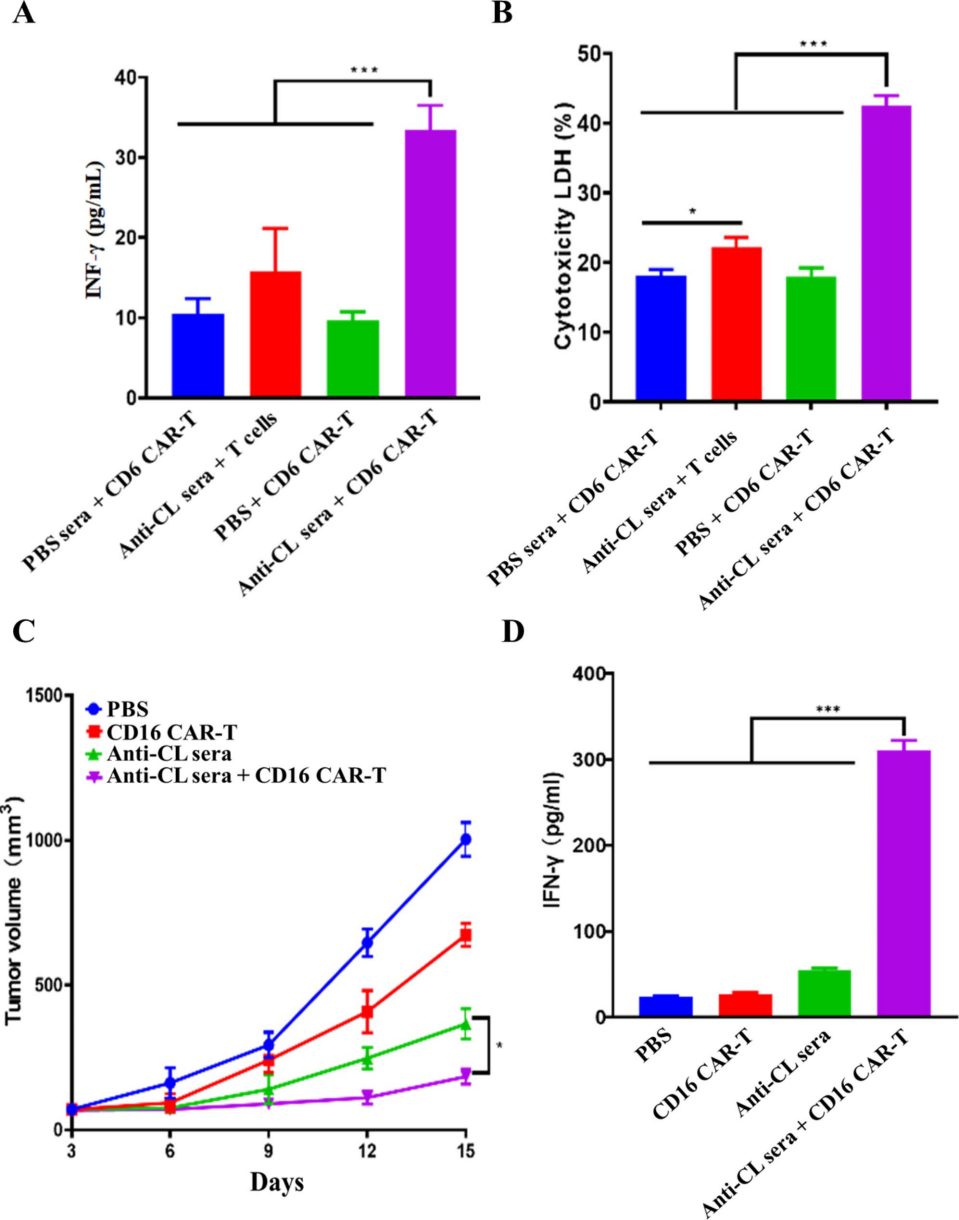
Synergistic anti-tumor activity of anti-CL sera with CD16 Car-T. An INF-γ secretion in cell culture media. (**B**) Cytotoxic effects on B16F10 tumor cells. (**C**) Tumor growth curves. (**D**) INF-γ secretion in sera. The data were presented as means ± s. d. Statistical significance was calculated by one-way analysis of variance (ANOVA). (n = 5), *, P < 0.05; ***p < 0.001

CD16 (FcγRIIIa) is a transmembrane FC-receptor expressed on large granular lymphocytes and involved in antibody-dependent cell-mediated cytotoxicity (ADCC). Immunization of CL is able to induce specific anti-melanoma humoral responses. We were thereafter interested in investigating whether CD16 CAR-T cells is able to enhance anti-tumor activity of anti-CL sera through CD16 CAR-mediated ADCC? The cytoplasmic domain of CD3ζ chain was then fused with extracellular domain of mouse CD16 molecule to construct chemiric antigen receptor (CAR) of CD16 (Fig. S12). Lentivirus was used to deliver CD16 CAR-expressing gene into isolated T cells and gene transduction efficiency was determined based on the expression of CD16 CAR in collected T cells through flow cytometry analysis (Fig. S13). Anti-CL sera were thereafter incubated with CD16 CAR-transfected T cells to detect the secretion of INF-γ in cell culture media. CD16 CAR-transfected T cells treated with sera isolated from PBS-treated mice, anti-CL sera-treated T cells (without CD16 transfection) and PBS-treated CD16 CAR-transfected T cells were used as control. The obtained results showed that, compared to CD16 CAR-transfected T cells treated with PBS, similar secretion of INF-γ was found in CD16 CAR-transfected T cells treated with sera isolated from PBS-treated mice. In contrast, increased secretion of INF-γ was significantly found in anti-CL sera-incubated CD16 CAR-transfected T cells, suggesting that, sera isolated from PBS-treated mice were not able to enhance INF-γ secretion and anti-CL sera were able to up-regulate INF-γ secretion in CD16 CAR-transfected T cells (Fig. 6A). However, INF-γ secretion in T cells treated with anti-CL sera were similar to those in CD16 CAR-transfected T cells treated with PBS, indicating that CD16 molecule played a central role in anti-CL sera-mediated INF-γ secretion and anti-CL sera were not able to up-regulate INF-γ secretion without CD16 transfection (Fig. 6A). The obtained results confirmed that anti-CL sera were able to interact with CD16 CAR expressed on the transfected T cell membrane surface, and activate CD16 CAR-mediated downstream signaling, which ultimately leads to the increased secretion of INF-γ. In addition, the remarkable up-regulation of INF-γ secretion revealed that CD16 CAR was successfully designed and constructed, which was able to not only interact with antibodies contained in anti-CL sera, but also mediate up-regulation of INF-γ secretion through designed intracellular CD3ζ-associated signaling pathway.

B16F10 cells were then treated with the mixture of the sera isolated from PBS-treated mice and CD16 CAR-T cells, the mixture of anti-CL sera and T cells, PBS-treated CD16 CAR-T cells and the mixture of anti-CL sera and CD16 CAR-T cells, respectively. Cell cytotoxicity was measured by using LDH-cytotoxicity assay kit. The observed data demonstrated that B16F10 cells treated with the mixture of the sera isolated from PBS-treated mice showed a similar cell cytotoxicity than the B16F10 cells treated with PBS-treated CD16 CAR-T cells, indicating that the sera isolated from PBS-treated mice had no anti-B16F10 tumor synergistic effects with CD16 CAR-T cells. In contrast, significant cell cytotoxicity was observed in B16F10 cells upon treatment with the mixture of anti-CL sera and CD16 CAR-T cells. The data suggested that the anti-CL sera had anti-B16F10 tumor synergistic effects with CD16 CAR-T cells (Fig. 6B). However, anti-B16F10 tumor effects of anti-CL sera was dramatically reduced while B16F10 tumor cells were treated with the mixture of anti-CL sera with T cells, which were not expressed CD16 CAR, suggesting a pivotal role of CD16 CAR in anti-B16F10 tumor synergistic effects of CD16 CAR-T cells with anti-CL sera (Fig. 6B). The data of cell cytotoxicity is consistent with the above INF-γ measurement assay, suggesting that anti-B16F10 tumor synergistic effects of CD16 CAR-T cells with anti-CL sera were realized through artificial CD16 CAR-mediated ADCC approach.

Anti-B16F10 tumor synergistic effects of CD16 CAR-T cells with anti-CL sera were further investigated by in vivo assay. Xenograft melanoma mice were treated by PBS, CD16 CAR T cells, anti-CL sera and the mixture of anti-CL sera and CD16 CAR T cells, respectively. The results showed that reduced tumor growth was not only observed in CD16 CAR-T cells-treated mice, but also in anti-CL sera-treated mice. However, the highest tumor growth inhibition was detected in mice treated with mixture of anti-CL sera and CD16 CAR T cells, which is correlated with the synergistic anti-B16F10 tumor effects of anti-CL sera with CD16 CAR T cells via ADCC approach (Fig. 6C).

The sera were collected from treated mice to detect the secretion of INF-γ in sera. The data showed that the secretion of INF-γ in sera of CD16 CAR-T cells-treated mice was similar to those from PBS-treated mouse sera. Remarkably increased secretion of INF-γ was found in sera of mice treated with the mixture of anti-CL sera and CD16 CAR-T cells, which indicated that CD16 CAR-T cell was not able to up-regulate INF-γ secretion without the presence of anti-CL sera, which is correlated with CD16 CAR-mediated up-regulation of INF-γ secretion via the expected ADCC pathway upon its interaction with antibodies presented in anti-CL sera (Fig. 6D). In addition, the slightly increased INF-γ secretion was detected in anti-CL sera-treated mice (Fig. 6D), suggesting that antibodies in anti-CL sera were probably able to interact with few endogenous immune cells that express CD16 molecule, which led to a modest up-regulation of INF-γ secretion in sera. However, the detailed mechanisms are still remained to be elucidated.

## Conclusion

In the current study, CMG and PS were used as nanomaterials to efficiently encapsulate CpG ODN and construct nano-adjuvant CNPs with a good biocompability. It is well known that DC cells play a key role in coupling innate to adaptive immune response. We further found that CNPs was not only able to targetedly deliver CpG ODN into DC cells and notably trigger DC activation and maturation, but also enhance anti-tumor activity of anti-PD1 antibody by modifying tumor environment via triggering the remarkable infiltration of CD3CD8^+^ T cells into tumor tissues. The obtained data next demonstrated that the mixture of CTL and neoantigen peptides represented the most effective tumor-derived antigen resources which were not only able to induce potent tumor-specific CTL responses, but also strong tumor-specific humoral immune responses in the presence of CNPs, which efficiently inhibited tumor cell growth in vitro and in vivo. We further found that CTL-induced humoral immune responses were able to specifically interact with CD16 CAR-T cells and dramatically suppressed tumor cell growth via ADCC pathway. Overall, our data demonstrated that CNP has a great potential to be a robust biocompatible adjuvant for anti-tumor vaccine generation by mixing with tumor-derived oncogenic antigens of CTL and neoantigens, which is able to be simultaneously used with CD16 CAR-T for vaccine/CAR-T cells-based synergistic targeted anti-tumor immune therapy. The generation of CD16 CAR-T in the present study represented an universal immune cell-based therapeutic approach by simultaneously used with vaccine for vaccine-induced tumor-specific antibodies-directed targeted immune T cell-based anti-tumor immunotherapy.

## Materials and methods

### Materials

CPG-ODN 1826 was used as TLR9-recognizing adjuvant and purchased from Sangon Biotch (Beijing, China). Its sequence is: TCCATGACGTTCCTGACGTT. Cy-5 fluorescence labeled CpG ODN 1826 were purchased from Sangon (Shanghai China). CMG and PS were obtained from Sigma-Aldrich (St. Louis, MO, USA).

### Cell lines and animals

Murine cancer cell line B16F10 and 4T1 were purchased from the National Infrastructure of Cell Line Resource (Beijing China). C57BL/6 (6–8 weeks old) female mice were purchased from the Vital River Laboratory Animal Technology Co., Ltd. (Beijing, China). All animal procedures were reviewed and ethically approved by the Institute of Basic Theories of Chinese Medicine, China Academy of Chinese Medical Sciences committee and authority for animal protection (Approval No: 20,190,901,063).

### Preparation and characterization of nanocomplexes

Nanocomplexes were generated by mixing CMG with various mass ratios of PS in aqueous solutions. Briefly, PS (1 mg/mL) and CMG (2 mg/mL) were separately dissolved in distilled water. Different volumes of PS solution was slowly dropwise added into CMG solution with or without CpG-ODN at room temperature with constant stirring. The nanoparticles without CpG-ODN was abbreviated as name NP and CpG-ODN-encapsulated nanocomplexes was abbreviated as name CNPs. The obtained nanocomplexes were thereafter harvested by centrifugation at 12,000 g for 15 min and resuspended with distilled water after washing twice. Lyophilized-nanocomplexes was weighted to evaluate product yields.

The morphologies of nanocomplexes were negatively stained with 3% uranyl acetate solution and visualized by transmission electron microscopy (TEM) (Tecnai G2 20 S-TWIN, FEI Company, Philips). The size and zeta potential of generated nanoparticles were analyzed by dynamic light scanning (DLS) (Malvern Instruments, UK).

### Effects of mass ratio of CpG ODN on entrapment efficiency

The Cy5-CpG fluorescent labelled nanoparticles were prepared and centrifuged at 5000 g for 15 min. Flow-through was analyzed using 2µL of sample on a Nanodrop Spectrophotometer, ND-1000 (Thermo Scientific, Wilmington, DE), measuring the absorbance wavelength. Concentration of CpG in flow-through (unencapsulated) was calculated using Beer-Lambert law (A = ε*b*c), with ε = 5,367.7 (M^− 1^ cm^− 1^), calculated from a standard curve of CpG. Final encapsulated concentration was calculated by subtracting the concentration in flow-through from the known concentration used for hydration.

### DAN electrophoresis

CNPs containing 500 µg CpG ODN and CpG ODN solution were dissolved in 1 mL of 1640 containing 50% serum and incubated at 37 °C for 24 h. Samples were taken at different time intervals. The CpG content was observed by gel imager after electrophoresis in 2% agarose gel.

### Cell cytotoxicity assay

B16F10 were seeded at a density of 2 × 10^4^ cells per well in 96-well plates and cultured at 37 ℃ with 5% CO2 for 12 h before evaluation. The culture media were removed and then replaced by 200 ml of RPMI 1640 supplemented with 10% fetal bovine serum containing (HUVEC cell culture media used HUV-EC-C of Pricella company) various equivalent concentrations of CNPs from 50 µg/mL to 400 µg/mL (the concentrations were represented by the concentrations of CpG ODN) for 48 h. Control cells were cultured in the same conditions without nanoparticles treatment. Cell viability was determined by using CCK-8 assay according to manufacturer’s instructions (Dojindo, Japan). The percentage of the viable cells was calculated using the following formula: Viability % = A - B/C - B × 100%, where A represents the absorbance of test, B represents the absorbance of blank (medium), C represents the absorbance of control (cells). Absorbance at 450 nm was detected with TECAN Infinite M200 microplate reader (Tecan, Durham, USA).

### Cellular uptake efficiency of CNPs in BMDCs

BMDCs were established by culturing bone marrow cells isolated from the femurs of C57BL/6 mice in RPMI 1640 (Gibco, USA) medium supplemented with 10% FBS (Gibco, USA), 50 µM β-mercaptoethanol (Sigma-Aldrich, USA), 1% penicillin/streptomycin (Gibco, USA), 20 ng/ mL GM-CSF and 5 ng/mL IL-4 (Peprotech, USA). On day 3 the media were all replaced and on day 6 the media were half replaced. Collected immature 2.5 × 10^5^ of BMDCs were put into confocal dishes (NEST, China) for 24 h at 37℃ with 5% CO_2_ on day 7. BMDCs were then incubated with Cy-5-labeled CpG ODN, and CNPs for 24 h. After washing with PBS for three times, cells were fixed by 4% paraformaldehyde for 30 min followed by labeling of 4’, 6-diamidine-2’-phenylindole dihydrochloride (DAPI, Thermo Fisher, USA) for 10 min. The cells were subsequently washed 2 times with PBS and photographed by using confocal laser scanning microscopic (CLSM, Carl Zeiss, USA).

### BMDCs activation, maturation and cytokine production

Established BMDCs were collected and plated at 2 × 10^6^ cells per well in 24-well plates and incubated 12 h later with different formulations, including PBS, NPs, CpG ODN and CNPs and continuously cultured in complete media for 24 h at 37℃ with 5% CO_2_. Treated-cells were harvested and blocked with anti-CD16/32 antibody (Biolegend, USA) at 4 ℃ for 5 min and stained with anti-CD11c, anti-MHC II anti-CD80 and anti-CD86 antibodies (Biolegend, USA). The maturation of BMDCs was analyzed by flow cytometry.

### In vivo examination of anti-tumor effects of CNPs and anti-PD1 antibody

To establish the tumor model, C57BL/6 mice were injected subcutaneously with 2 × 10^5^ B16F10 cells suspended in 100 ml of PBS on the right flank. When tumor size reached around 100 mm^3^, the mice were randomly assigned to five groups (n = 5 for each group) and subcutaneously injected with PBS, NP (empty nanoparticle), CpG ODN and CNPs on days 0, 7, 14. Anti-PD1 antibody (aPD1) was administrated by intraperitoneal injection at above indicated time points. The used doses of aPD1, NP, CpG ODN and CNPs were 100.0 µg/mouse, 25.0 µg/mouse, 20.0 µg/mouse and 20.0 µg/mouse, respectively. Tumor growth was subsequently measured every 3 days with a caliper and tumor volumes were calculated by blinded investigators using the formula V = 0.5 × length × width^2^. On day 21 of the final immunization, mice were sacrificed for further analysis.

### Isolation and evaluation of tumor-infiltrating T cells

Tumor tissues were harvested and subsequently cut into small pieces and then placed in RPMI 1640 media containing 0.4 mg/mL collagenase D (Roche, Switzerland) and 0.2 mg/ mL DNase (Solarbio, China) for 30 min at 37 ℃ with gentle shaking for CD3^+^CD4^+^ and CD3^+^CD8^+^ T cell isolation. The cell suspension was passed through a 70 μm strainer and then leukocytes were obtained with lymphocyte separation medium by density gradient centrifugation (DAKEWE, China). The isolated cells were further stained with anti-CD3, anti-CD8a and anti-CD4a antibodies (Biolegend, USA) for flow cytometric analysis.

### In vivo CD8^+^ T elimination and tumor growth assay

2 × 10^5^ B16F10 cells suspended in 100 ml of PBS were subcutaneously injected in C57BL/6 mice on the right flank. When tumor sizes reached around 100 mm^3^, the mice were randomly assigned to five groups (n = 5 for each group) and subcutaneously injected with CNPs (20.0 µg/mouse) on days 0, 7 and 14. Anti-PD1 antibody (aPD1) and anti-CD8 antibody (Biolegend, USA, 100.0 µg/mouse) was simultaneously administrated by intraperitoneal injection at above indicated time points. Tumor growth was subsequently measured every 3 days with a caliper and tumor volumes were calculated by blinded investigators using the formula V = 0.5 × length × width^2^. On day 21 of the final immunization, mice were sacrificed for further analysis.

### Elisa analysis

The secretion of IL-6, IL-12p40 in the BMDC-cultured supernatants was analyzed by ELISA kits for Interleukin 6 (IL6) and Interleukin 12 p40 (Biolegend, USA), respectively, based on manufacturer’s protocols. The presence of IFN-γ and TNF-α in the sera harvested from mice of each group was detected by ELISA Kit for Tumor Necrosis Factor Alpha (TNFa) and Interferon Gamma (IFN-γ) according to the enclosed manufacturer’s instructions (Biolegend, USA).

High sensitive Mouse IgG ELISA Kit (Bethyl, USA) was used to quantitatively analyzed IgG antibodies presented in sera of CL-immunized mice according to the enclosed protocols, in which TCL (10 µg/mL) was used as tumor antigen resources and coated on the plate surfaces for ELISA analysis.

### In vivo anti-tumor assay of tumor-derived antigens-based vaccines

CNPs was separately mixed with TCL, neoantigens and the mixture of TCL and neoantigens to generate various CNPs-adjuvanted tumor-derived antigens-based vaccines named as CL, CN and CLN, respectively, in which the doses of used CNPs, TCL and neoantigens were 20.0 µg, 50.0 µg and 50.0 µg, respectively. C57BL/6 mice were injected subcutaneously with 2 × 10^5^ B16F10 cells on the right flank. When tumor sizes reached about 100 mm^3^, mice were randomly assigned into eight groups (n = 6 for each group) and subcutaneously injected every 7 days for 3 times with PBS, lysate, neoantigens, LN, CNPs, CL. CN and CLN, respectively. Tumor growth was subsequently measured every 3 days with a caliper and tumor volumes were calculated by blinded investigators using the formula V = 0.5 × length × width^2^. On day 21 of the final immunization, mice were sacrificed for further analysis.

### Body weight and survival rate analysis

Animal body weight was measured every 3 days for 21 days. The survival rate of mice treated with various substrates was also analyzed and calculated by dividing the number of surviving mice at different time points of post-treatment with the total number of mice before treatment.

### Elispot

To determinate the production of IFN-γ, the splenocytes were collected and cultured in media containing the mixture of TCL and neoantigens (total: 20 µg/ml) for 48 h at 37 ℃ in ELISpot plates (Mabtech, Sweden). The IFN-γ production was measured by ELISpot kit according to the provided protocols (Biolegend, USA). The numbers of IFN-γ spots were analyzed using an AID-ELISpot-Reader (AID, Strassberg, Germany).

### In vitro CTL assay

The T cells were collected from isolated spleens for in vitro CTL analysis. The ratios of harvested T cells to target tumor cells were 50:1 and 100:1, respectively. The CTL activity was detected by lactate dehydrogenase (LDH) assay (Dojindo, Kumamoto, Japan). Specific lysis was calculated with the following formula: [(experimental LDH release - effector cells spontaneous LDH release – target cells spontaneous LDH release) / (target cells maximum LDH release – target cells spontaneous LDH release)] × 100%.

### Memory T cell analysis

For memory T cell analysis, the splenocytes were isolated and primed with TCL overnight and continuously cultured for additional 48 h at 37 ℃. The treated cells were harvested and stained with anti-CD4-APC, anti-CD8-PE, anti-CD44-FITC and anti-CD62L-Percp antibodies. The proportion of effector memory T cells (CD44highCD62Llow) and central memory T cells (CD44highCD62Lhigh) were detected by flow cytometer (BD Biosciences, San Jose, CA).

### In vitro and in vivo anti-tumor assays for collected sera

To examine the in vitro anti-tumor effects of collected sera, 5 × 10^3^ tumor cells were cultured in 96-well plates and treated with 1.0 µl, 5.0 and 10.0 µl of collected sera for 48 h, separately. PBS-treated cells were used as control. Anti-tumor activity was quantitatively evaluated by LDH cytotoxicity assay kit based on the provided protocols. To investigate the in vivo anti-tumor effects of collected sera, 2 × 10^5^ B16F10 cells and 1 × 10^6^ 4T1 cells were subcutaneously injected in C57BL/6 mice on the right flank. When tumor size reached about 100 mm^3^, the mice were randomly assigned to 4 groups (n = 5 for each group) and intraperitoneally injected with 100 µl of anti-CL sera and anti-PBS sera, respectively once a week for two weeks. The tumor volumes were monitored and recorded every three days.

### Preparation of CD16 CAR-T

The construction of CD16 CAR was achieved by fusing extracellular domain of mouse CD16 with transmembrane domain of CD28 and the signaling elements from CD3-ζ, which mediated T cell signaling. CD16 CAR-encoding gene was subcloned in pLVX-Puro vector by using restriction sites XhoI and XbaI. For lentivirus preparation, 293FT cells were simultaneously co-transfected with CD16 CAR-encoding vectors and packaging construct plasmids as manufacturer’s instructions described (Clontech, USA). Supernatant was harvested from transfected 293FT cells 48 h following transfection, filtered using 0.45 μm sterile filters, and concentrated by ultracentrifugation using a Beckman SW32 rotor at 30,000 rpm at 4 °C. Media were aspirated and pellets were resuspended with PBS and stored at -80 °C. Various doses of recombinant lentivirus were used to determine the optimal dose for isolated T cell infection. The expression of CD16 CAR in infected T cells was eventually evaluated by anti-CD16/32- percpcy5.5 and anti-CD3-FITC antibodies staining via flow cytometry analysis (Biolegend, USA).

### In vitro and in vivo anti-tumor assays by using collected sera and CD16 CAR-T cells

To examine the in vitro synergistic anti-tumor effects of collected sera and CD16 CAR-T cells, 5 × 10^3^ B16F10 tumor cells were cultured in 96-well plates and treated with 5.0 µl collected sera and 1 × 10^5^ of CD16 CAR-T cells for 48 h, separately. Three wells were used for each sample and the examination was repeated twice. Anti-tumor activity was quantitatively evaluated by LDH cytotoxicity assay kit. To investigate the in vivo synergistic anti-tumor effects of collected sera and CD16 CAR-T cells, 2 × 10^5^ B16F10 cells and 1 × 10^6^ 4T1 cells were subcutaneously injected in C57BL/6 mice on the right flank. When tumor size reached about 100 mm^3^, the mice were randomly assigned to 4 groups (n = 5 for each group). 100 µl of anti-CL sera and anti-PBS sera were intraperitoneally injected and 2 × 10^6^ CD16 CAR-T cells were administrated by tail vein injection. The injection was carried out once a week for two weeks. The tumor volumes were monitored and recorded every three days.

### Hematoxylin and eosin staining

Major organs were harvested and fixed in 10% formalin and used for hematoxylin and eosin staining at the Peking University Health Science Center. The histological sections were observed and imaged under optical microscopy.

### Statistical analysis

All data are determined as mean ± SD and all experiments were performed at three times. Statistical significance of the data was considered by the one-way analysis of variance (ANOVA). Values of *p < 0.05 and **p < 0.01 were considered statistically significant.

## Electronic supplementary material

Below is the link to the electronic supplementary material.

Supplementary Material 1

Supplementary Material 2

Supplementary Material 3

Supplementary Material 4

## Data Availability

Without restrictions.

## Abbreviations

PS: Protamine sulfate
CpG ODN: CpG oligodeoxynucleotides
DCs: Dendritic cells
CTL: Cytotoxic T lymphocyte
APCs: Antigen-presenting cells
TAA: Tumor-associated antigen
TSA: Tumor-specific antigens
TLR: Toll-like receptors
TNF-α: Tumor necrosis factor
IFN-γ: Interferon-γ
DAPI: 4′,6-diamidine-2′-phenylindole dihydrochloride
LDH: Lactate dehydrogenase
ELISPOT: Enzyme-linked immunoblot
TCL: Tumor cell lysate.
