# Supplementary material for: CD16 CAR-T cells enhance antitumor activity of CpG ODN-loaded nanoparticle-adjuvanted tumor antigen-derived vaccinevia ADCC approach
Source: J Nanobiotechnology. 2023 May 20;21:159. doi: 10.1186/s12951-023-01900-8 (PMC10199637; doi:10.1186/s12951-023-01900-8)
Supplement: Supplementary file 1 — Supplementary Material 1 [file 12951_2023_1900_MOESM1_ESM.docx]

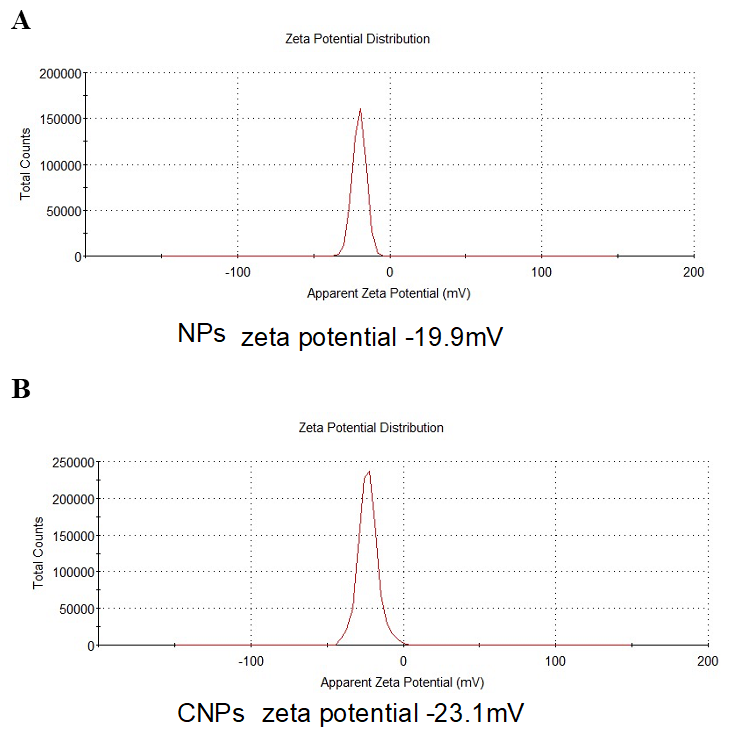


**Fig. S1** Zeta potential analysis. A zeta potential of NPs; B zeta potential of CNPs.


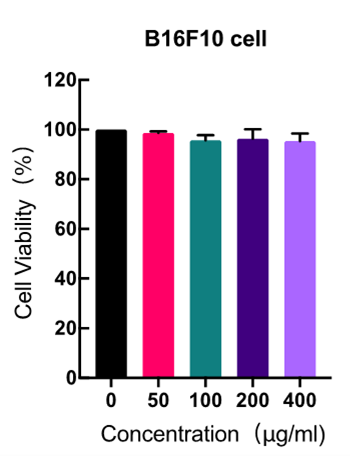


**Fig. S2** The cytotoxicity of CNPs in B16F10 cells.

**Fig. S3** The expressions of CD80 and CD86 in BMDCs treated by PBS, NP, free CpG ODN and CNP.

**Fig. S4** Lymph node targeting effects of mice treated with different components (free CpG, CNP) at a different time (6 h, 12 h, 24 h, 72 h after injection), CpG were modified with Cy5.


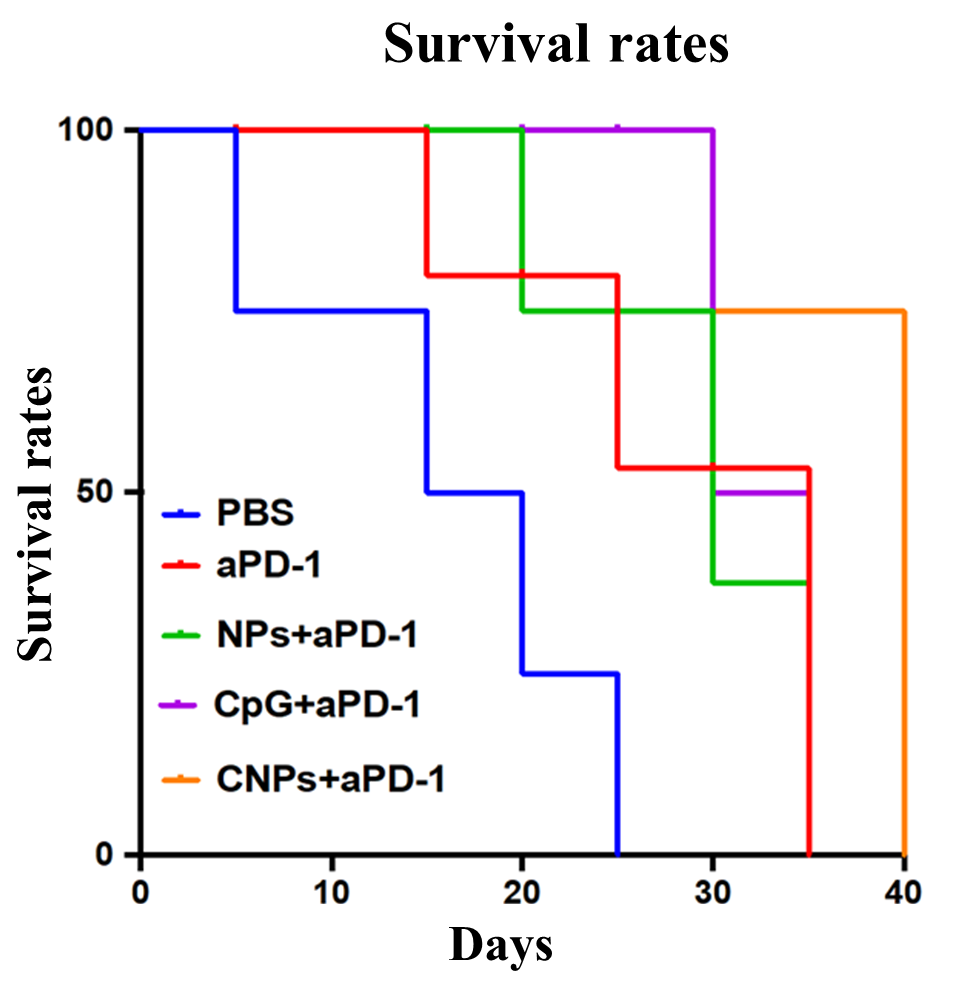


**Fig. S5** The survival rates of mice.


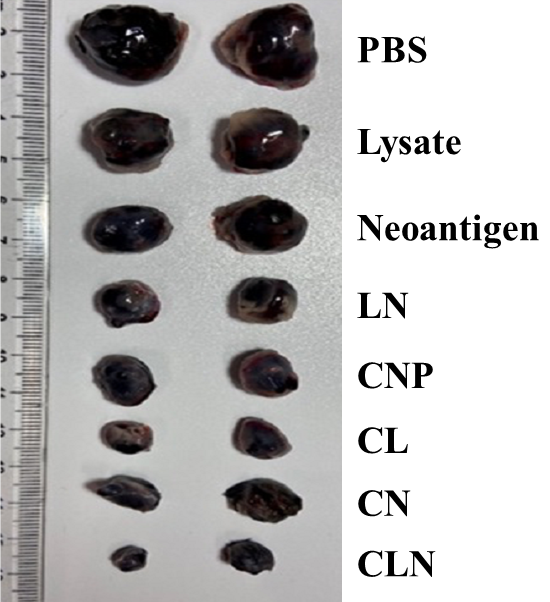


**Fig. S6.** Tumor sizes.


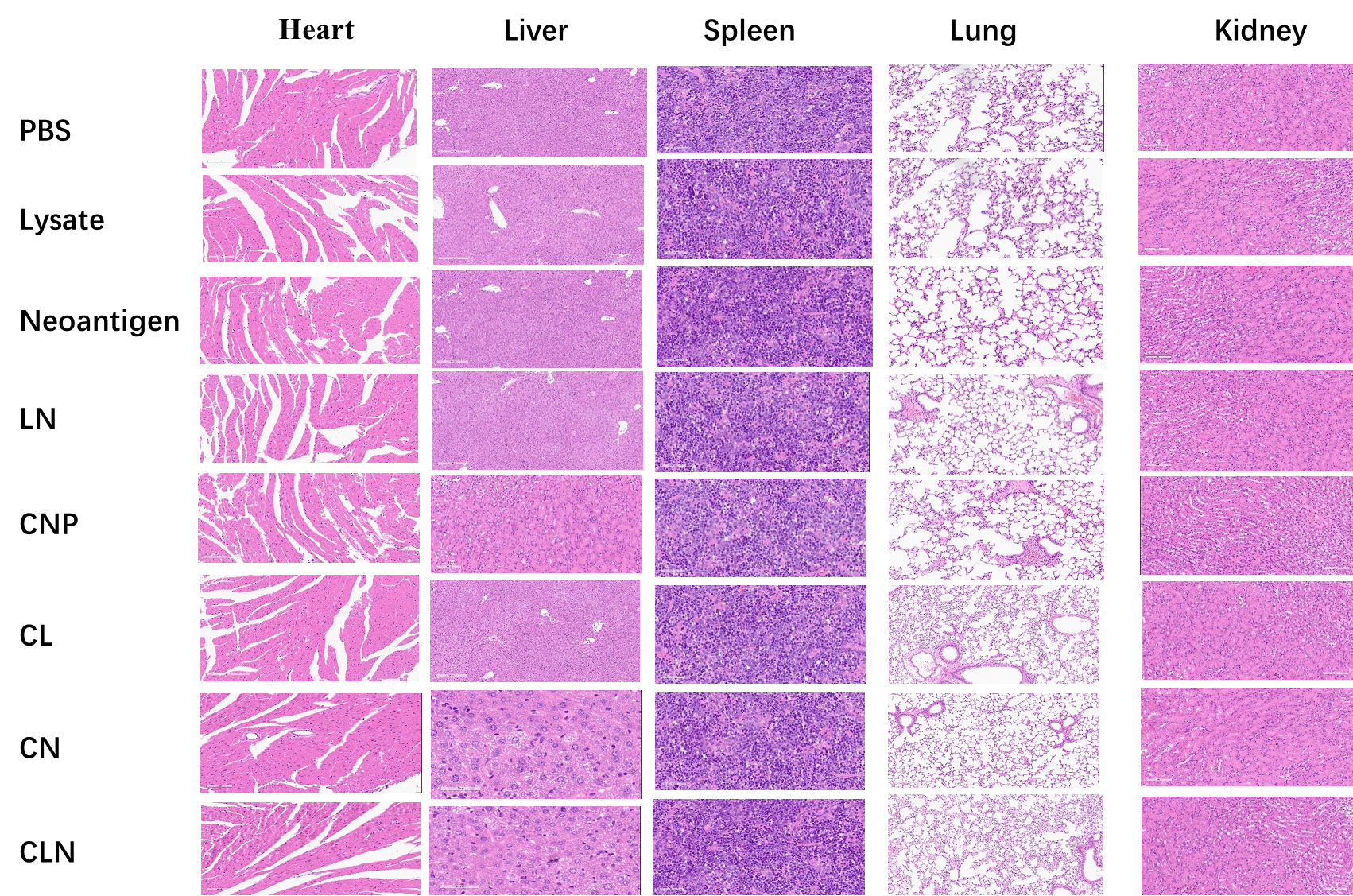


**Fig. S7** H&E staining of organs in various groups of mice.

**Fig. S8** Expressions of T cells surface markers CD4 and CD8.

**Fig. S9** Expressions of memory T cells surface markers CD44 and CD62L.

CD16-CAR sequence 5′- 3′
ATGTTCCAGAACGCCCACAGCGGCTCCCAGTGGCTGCTGCCCCCACTGACCATCCTGCTGCTGTTCGCCTTCGCCGACCGCCAGAGCGCCGCCCTGCCTAAGGCCGTGGTGAAGCTGGACCCACCCTGGATCCAGGTGCTGAAGGAGGACATGGTGACCCTGATGTGTGAGGGCACCCACAACCCCGGCAACAGCTCCACCCAGTGGTTCCACAACGGCCGGAGCATCAGGTCCCAGGTGCAGGCCAGCTACACATTCAAGGCCACCGTGAACGACAGCGGCGAGTACAGGTGCCAGATGGAGCAGACACGGCTGAGCGACCCCGTGGACCTGGGCGTGATCAGCGACTGGCTGCTGCTGCAGACCCCTCAGAGGGTGTTCCTGGAGGGCGAGACAATCACTCTGAGATGTCACTCTTGGAGAAATAAGCTGCTGAATAGAATCAGCTTCTTCCACAATGAGAAAAGCGTGAGATATCATCATTATAAGTCTAATTTTTCCATCCCAAAGGCTAATCACTCTCACTCCGGCGACTACTACTGTAAAGGAAGTCTGGGAAGCACACAGCACCAGTCCAAGCCAGTGACTATTACTGTGCAGGACCCTGCTACAACAAGCTCTATCTCCCTGGTGTGGTATCACACAATCGAGTTCATGTATCCTCCACCTTATCTTGACAATGAGAGGTCCAATGGTACCATCATTCATATTAAGGAAAAACACCTGTGTCACACCCAGAGCAGCCCCAAGCTGTTCTGGGCCCTGGTGGTGGTGGCCGGCGTGCTGTTCTGTTACGGCCTGCTGGTGACCGTGGCCCTGTGCGTGATCTGGACCAACTCCAGGCGGAACAGGGGCGGCCAGAGCGACTACATGAACATGACCCCTAGACGGCCTGGCCTGACCAGAAAGCCTTACCAGCCTTACGCCCCCGCCAGAGACTTCGCCGCCTACAGACCAAGGGCCAAGTTCAGCAGGAGCGCCGAGACCGCCGCCAACCTGCAGGACCCCAACCAGCTGTTTAACGAGCTGAACCTGGGCAGAAGGGAGGAGTTCGACGTGCTGGAGAAGAAGAGGGCCAGGGACCCAGAGATGGGCGGCAAGCAGCAGAGGAGGAGAAACCCTCAGGAGGGCGTGTACAACGCTCTGCAGAAGGACAAGATGGCCGAGGCCTACAGCGAGATCGGCACCAAGGGCGAGAGGAGGAGAGGCAAGGGCCACGACGGCCTGTTCCAGGGCCTGAGCACCGCCACCAAGGACACCTTTGACGCCCTGCACATGCAGACCCTGGCCCCTAGG

**Fig. S10** The sequence of CD16-CAR T.


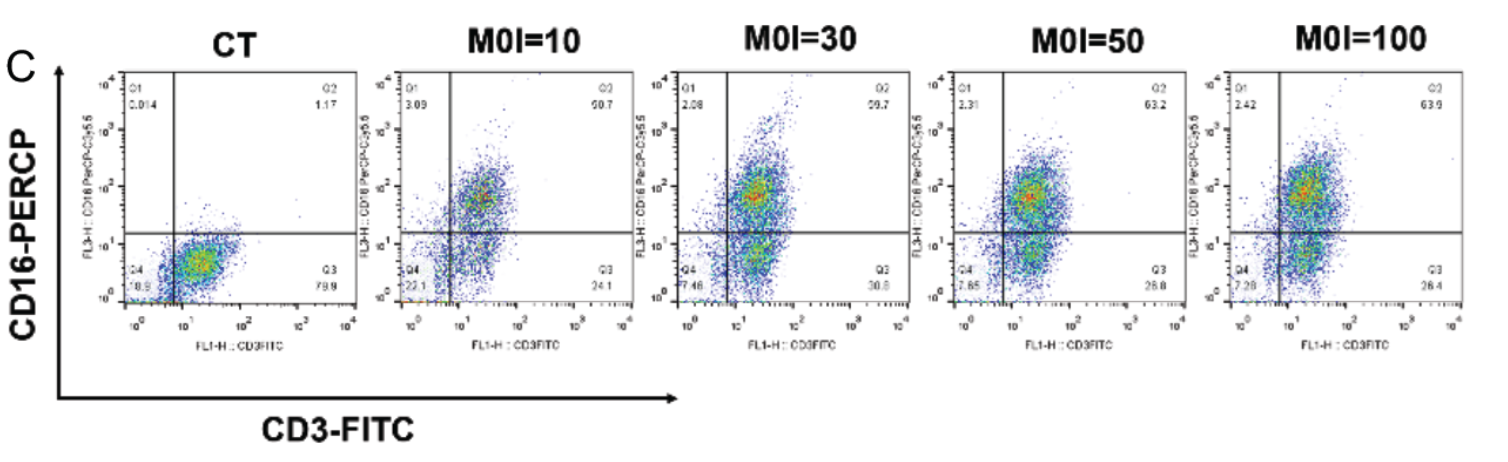


**Fig. S11** The expression of CD16 CAR was detected by flow cytometry analysis.
