# Supplementary material for: CD16 CAR-T cells enhance antitumor activity of CpG ODN-loaded nanoparticle-adjuvanted tumor antigen-derived vaccinevia ADCC approach
Source: J Nanobiotechnology. 2023 May 20;21:159. doi: 10.1186/s12951-023-01900-8 (PMC10199637; doi:10.1186/s12951-023-01900-8)
Supplement: Supplementary file 2 — Supplementary Material 2 [file 12951_2023_1900_MOESM2_ESM.docx]

**CD16 CAR-T cells Enhance Antitumor Activity of CpG ODN-Loaded Nanoparticle-Adjuvanted Tumor Antigen-Derived**

**VaccineVia ADCC Approach**

**Supporting data**

| **PS:CMG:CpG** | **1:2：0.1** | **1:2：0.05** | **1:2：0.0375** | **1:2：0.035** | **1:2：0.03** |
| --- | --- | --- | --- | --- | --- |
| **entrapment efficiency of CpG-ODN (EE) %** | 68.3 | 84．5 | 97.3 | 97.4 | 97.4 |

**Table.S1** Effects of mass ratio of materials on entrapment efficiency (EE).


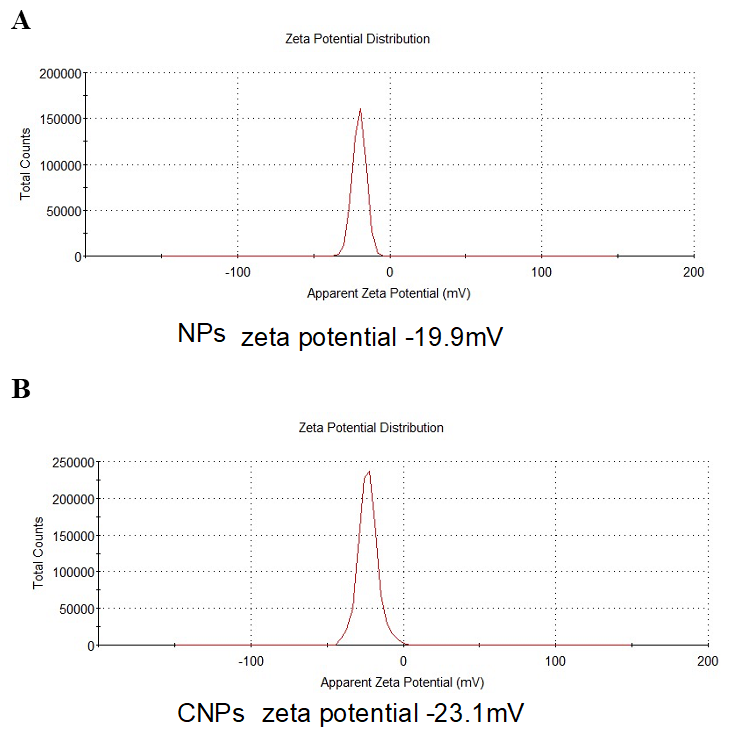


**Fig. S1** Zeta potential analysis. A zeta potential of NPs; B zeta potential of CNPs.


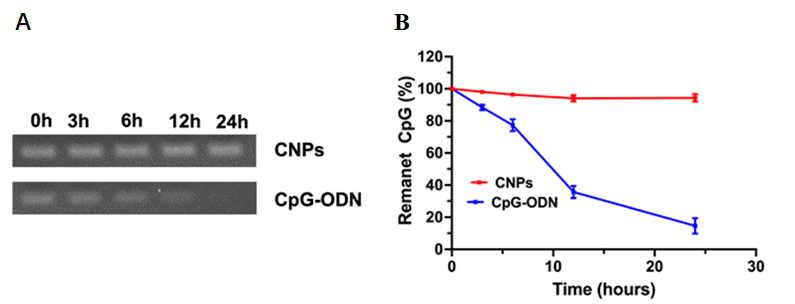


**Fig. S2** CpG ODN stability in serum. A Detection of CpG-ODN by electrophoresis. B The percentage of remained CpG-ODN at indicated time points by comparing the signal density of remained CpG-ODN with that at 0 hour.

**
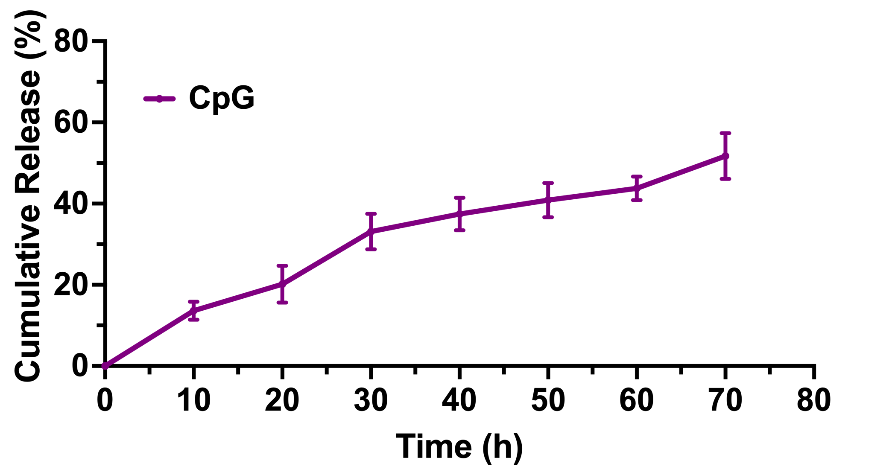
**

**Fig. S3.** In vitro release curve of CpG ODN from CNPs (n=3).


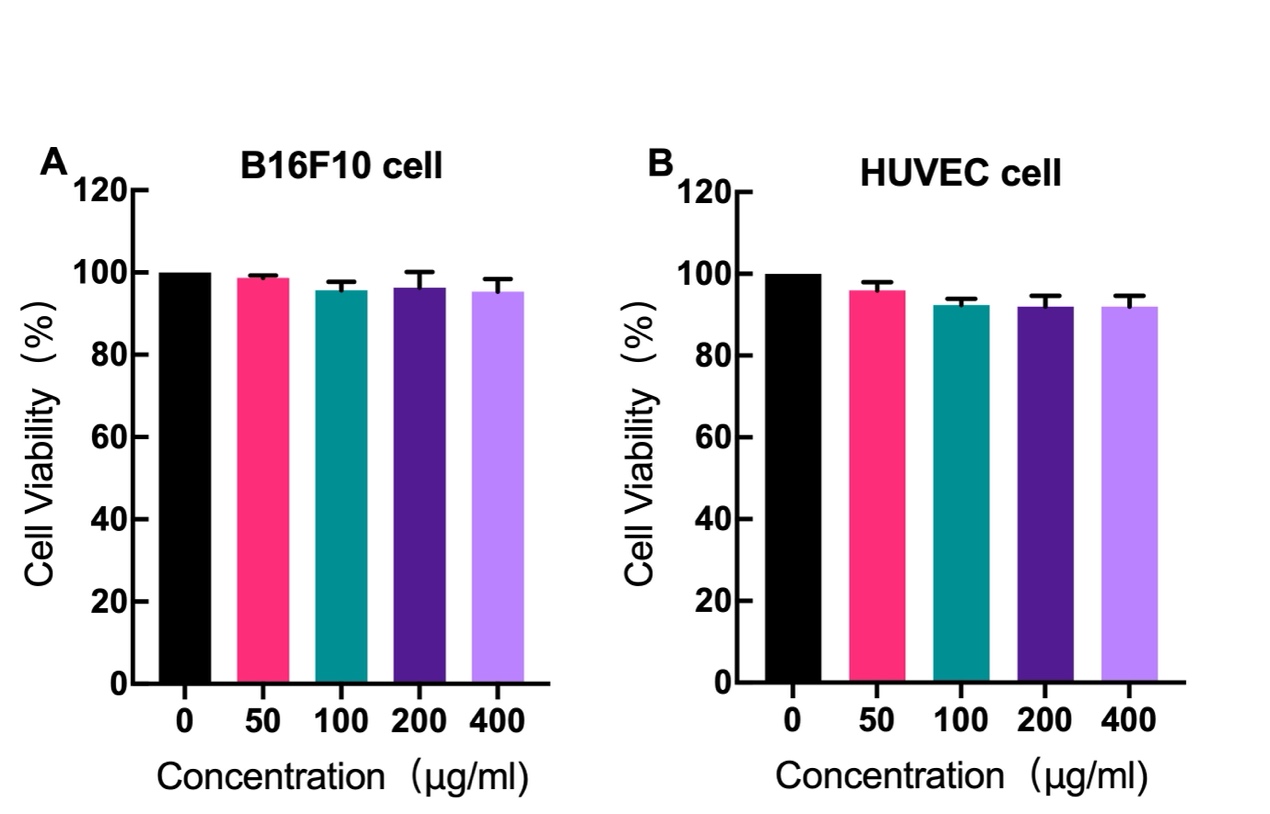


**Fig.** **S4** The cytotoxicity of CNPs in B16F10 cells and HUVEC cells.

**Fig. S5** The expressions of CD80 and CD86 in BMDCs treated by PBS, NP, free CpG ODN and CNP.


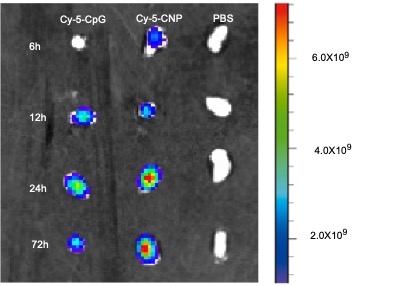


**Fig. S6** Lymph node targeting effects in mice treated with different components (free CpG ODN, CNP) at a different time points (6 h, 12 h, 24 h, 72 h after injection). CpG ODN was labeled with Cy5.


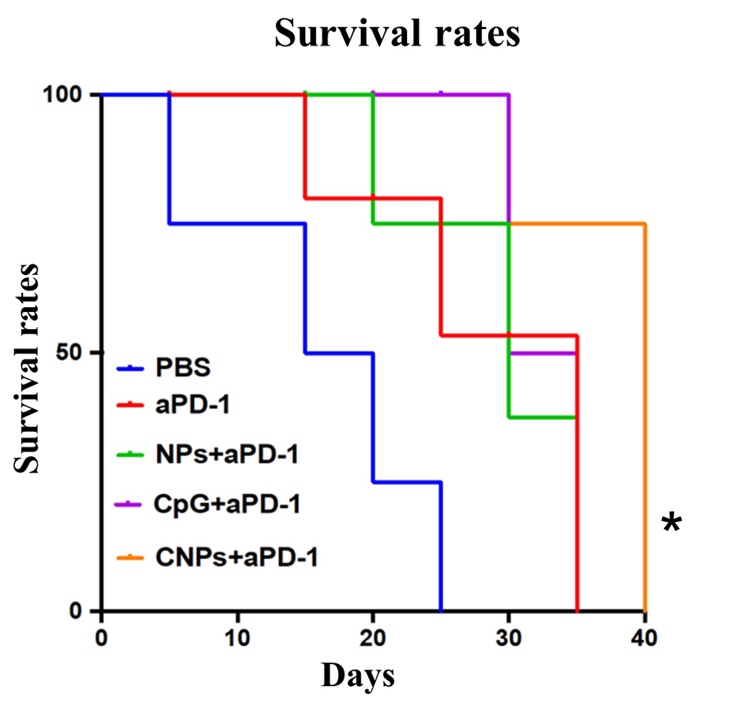


**Fig. S7** The survival rates of mice.


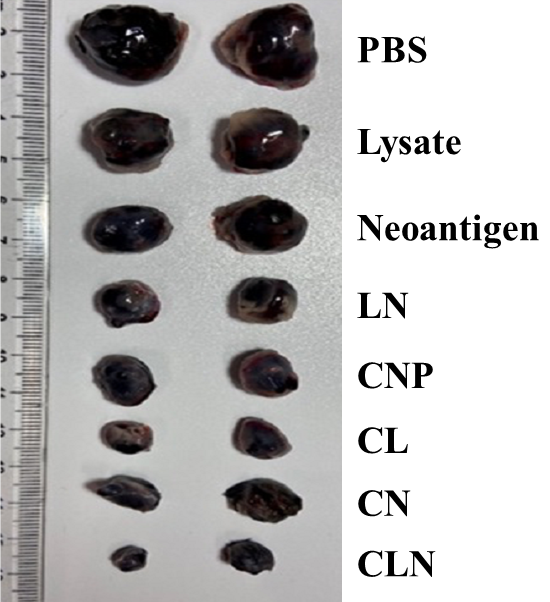


**Fig. S8** Tumor sizes.


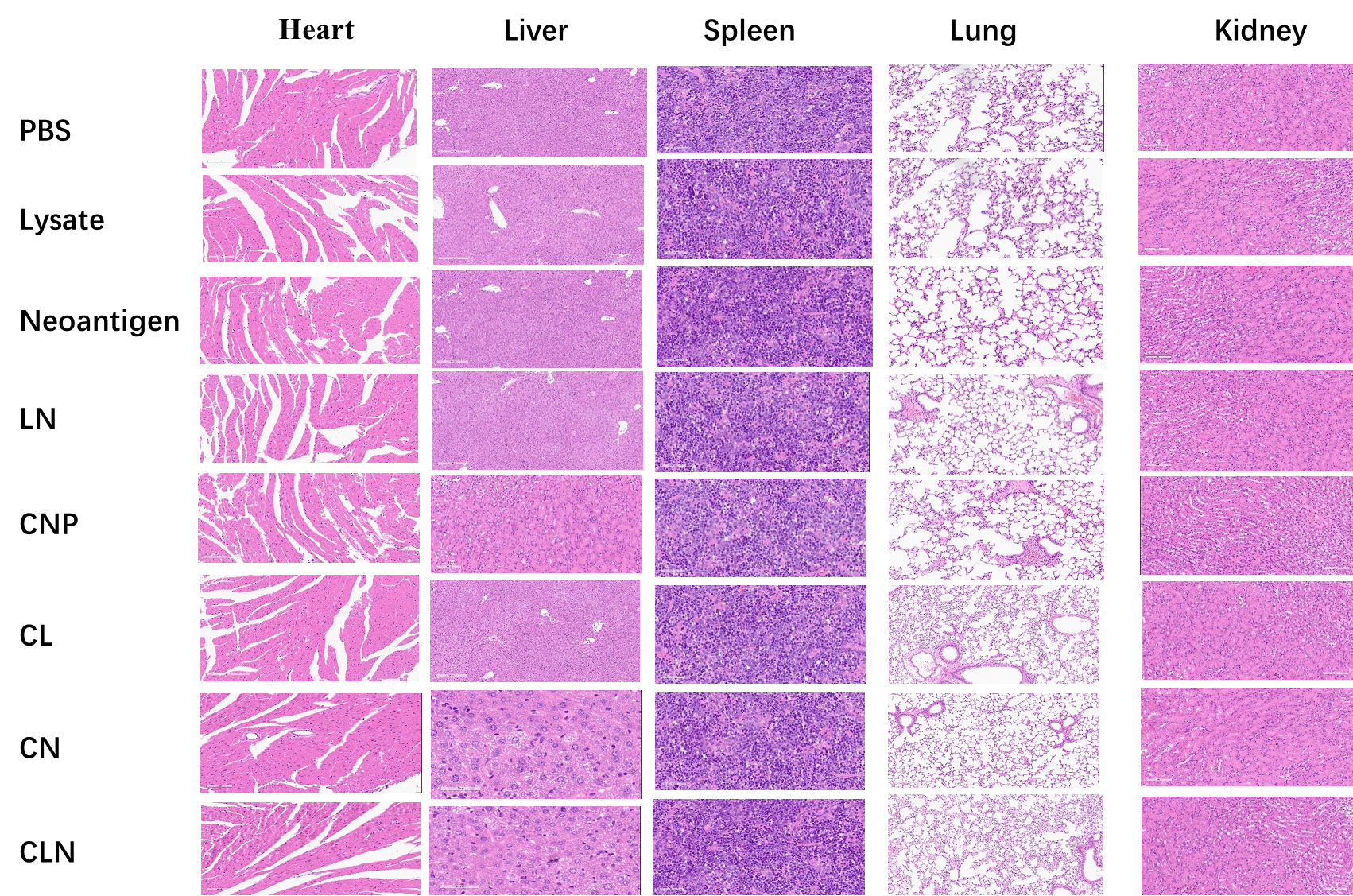


**Fig. S9** H&E staining of organs in various groups of mice.

**Fig. S10** Expressions of T cells surface markers CD4 and CD8.

**Fig. S11** Expressions of memory T cells surface markers CD44 and CD62L.

CD16-CAR sequence 5′- 3′
ATGTTCCAGAACGCCCACAGCGGCTCCCAGTGGCTGCTGCCCCCACTGACCATCCTGCTGCTGTTCGCCTTCGCCGACCGCCAGAGCGCCGCCCTGCCTAAGGCCGTGGTGAAGCTGGACCCACCCTGGATCCAGGTGCTGAAGGAGGACATGGTGACCCTGATGTGTGAGGGCACCCACAACCCCGGCAACAGCTCCACCCAGTGGTTCCACAACGGCCGGAGCATCAGGTCCCAGGTGCAGGCCAGCTACACATTCAAGGCCACCGTGAACGACAGCGGCGAGTACAGGTGCCAGATGGAGCAGACACGGCTGAGCGACCCCGTGGACCTGGGCGTGATCAGCGACTGGCTGCTGCTGCAGACCCCTCAGAGGGTGTTCCTGGAGGGCGAGACAATCACTCTGAGATGTCACTCTTGGAGAAATAAGCTGCTGAATAGAATCAGCTTCTTCCACAATGAGAAAAGCGTGAGATATCATCATTATAAGTCTAATTTTTCCATCCCAAAGGCTAATCACTCTCACTCCGGCGACTACTACTGTAAAGGAAGTCTGGGAAGCACACAGCACCAGTCCAAGCCAGTGACTATTACTGTGCAGGACCCTGCTACAACAAGCTCTATCTCCCTGGTGTGGTATCACACAATCGAGTTCATGTATCCTCCACCTTATCTTGACAATGAGAGGTCCAATGGTACCATCATTCATATTAAGGAAAAACACCTGTGTCACACCCAGAGCAGCCCCAAGCTGTTCTGGGCCCTGGTGGTGGTGGCCGGCGTGCTGTTCTGTTACGGCCTGCTGGTGACCGTGGCCCTGTGCGTGATCTGGACCAACTCCAGGCGGAACAGGGGCGGCCAGAGCGACTACATGAACATGACCCCTAGACGGCCTGGCCTGACCAGAAAGCCTTACCAGCCTTACGCCCCCGCCAGAGACTTCGCCGCCTACAGACCAAGGGCCAAGTTCAGCAGGAGCGCCGAGACCGCCGCCAACCTGCAGGACCCCAACCAGCTGTTTAACGAGCTGAACCTGGGCAGAAGGGAGGAGTTCGACGTGCTGGAGAAGAAGAGGGCCAGGGACCCAGAGATGGGCGGCAAGCAGCAGAGGAGGAGAAACCCTCAGGAGGGCGTGTACAACGCTCTGCAGAAGGACAAGATGGCCGAGGCCTACAGCGAGATCGGCACCAAGGGCGAGAGGAGGAGAGGCAAGGGCCACGACGGCCTGTTCCAGGGCCTGAGCACCGCCACCAAGGACACCTTTGACGCCCTGCACATGCAGACCCTGGCCCCTAGG

**Fig. S12** The sequence of CD16-CAR T.


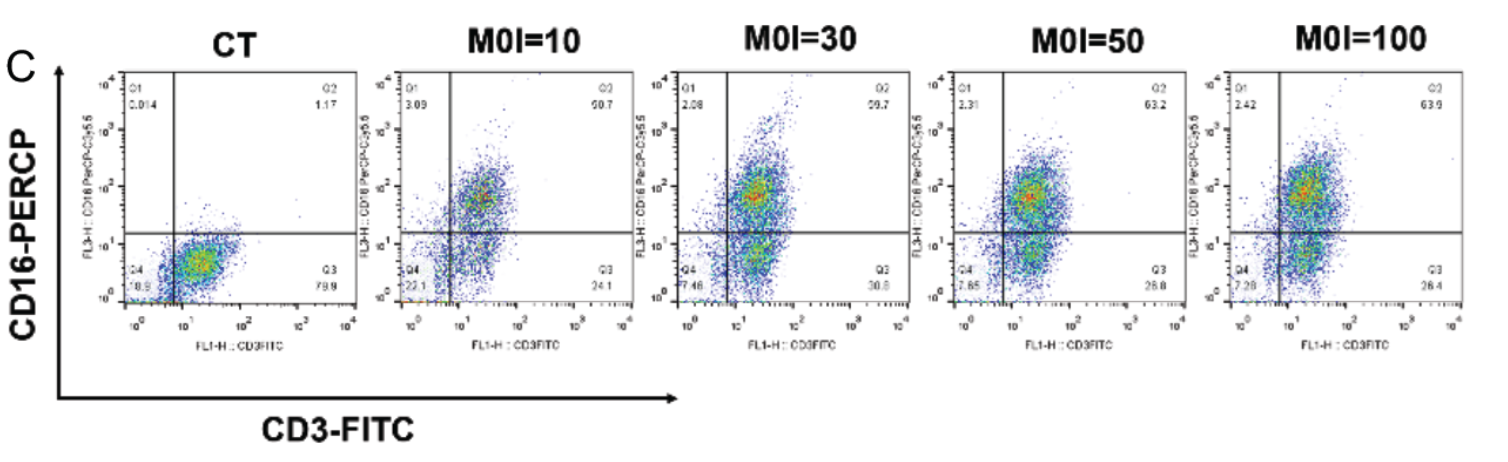


**Fig. S13** The expression of CD16 CAR was detected by flow cytometry analysis.
